# Molecular evolution and functional divergence of zebrafish (*Danio rerio*) *cryptochrome* genes

**DOI:** 10.1038/srep08113

**Published:** 2015-01-29

**Authors:** Chao Liu, Jia Hu, Chunxiang Qu, Lin Wang, Guodong Huang, Pengfei Niu, Zhaomin Zhong, Fashui Hong, Guanghui Wang, John H. Postlethwait, Han Wang

**Affiliations:** 1 Center for Circadian Clocks, Soochow University, Suzhou 215123, Jiangsu, China; 2 School of Biology & Basic Medical Sciences, Medical College, Soochow University, Suzhou 215123, Jiangsu, China; 3 School of Computer Science and Technology, Soochow University, Suzhou 215006, Jiangsu, China; 4 College of Pharmaceutical Sciences, Soochow University, Suzhou 215123, Jiangsu, China; 5 Institute of Neuroscience, 1254 University of Oregon, Eugene, OR 97403, USA

## Abstract

Cryptochromes function in animal circadian regulation. Zebrafish are known to have six *cryptochrome* (*cry*) genes but their evolutionary relationships are not yet fully resolved. Here, comparative genomic analyses revealed that a local duplication of ancestral chordate *Cry* occurred likely before the first round of vertebrate genome duplication (VGD); following two successive rounds of VGD and subsequent gene losses, coelacanths retained *cry1a*, *cry1b*, *cry2* and *cry3*; and following the third-round teleost genome duplication (TGD) and subsequent gene losses, zebrafish retained six *cry* genes, renamed as *cry1aa* (*zcry1a* in the old nomenclature), *cry1ab* (*zcry1b*), *cry1ba* (*zcry2a*), *cry1bb* (z*cry2b*), *cry2* (*zcry3*) and *cry3* (*zcry4*). Molecular evolutionary analyses suggested that zebrafish *cry* genes have evolved divergent functions, which is further supported by their distinct and rhythmic expression patterns as shown by both *in situ* hybridization and quantitative real-time PCR. Systematic cell transfection assays divided six Cry proteins into repressive Cry1aa, Cry1ab, Cry1ba and Cry1bb, and non-repressive Cry2 and Cry3. Cry2 is non-repressive because it lacks an effective protein-protein interaction domain although it does possess a nuclear localization signal (NLS) motif, whilst Cry3 lacks both an NLS motif and a protein-protein interaction domain. These findings provide a better understanding of evolution of zebrafish *cry* genes.

Cryptochrome proteins belong to the DNA photolyase/cryptochrome family, which is classified into five subfamilies according to molecular phylogenetic analyses and functions: class I cyclobutane pyrimidine dimer photolyase, class II cyclobutane pyrimidine dimer photolyase, plant CRY, animal CRY including (6–4) photolyases (6-4PHR), and cryptochrome DASH^1^,^2^. All members of this family share an N-terminal phytolyase homology (PHR) domain that can bind to the flavin adenine dinucleotide (FAD) cofactor and a light-harvesting chromophore^1^,^2^. CRY proteins were originally discovered in plants and shown to mediate blue light-dependent growth and development^3^,^4^. Subsequently, CRY proteins were shown to play roles in circadian regulation through light-dependent or light-independent mechanisms in animals^5^,^6^,^7^,^8^.

Two mouse CRY proteins (mCRY1 and mCRY2) are the canonical components of the circadian clock that controls daily rhythms of physiology and behavior^9^,^10^. The circadian clock oscillates autonomously, and its underlying mechanism is a self-sustained transcriptional/translational feedback loop composed of activators and repressors^9^. In the mouse circadian feedback loop, mPER and mCRY form a heterodimer that inhibits transcription mediated by another heterodimer formed by activators CLOCK and BMAL1. The mCRY proteins possess much greater light-independent inhibitive abilities than any of the three mPER proteins^11^,^12^,^13^. In the fruit fly (*Drosophila melanogaster*), the transcriptional activators are CLOCK and CYCLE (the insect ortholog of BMAL) and the inhibitors that block these activators are PER and TIM^14^. The fly CRY protein does not function in the core feedback loop, but instead binds TIM and represses the inhibitory action of the PER:TIM heterodimer in a light-dependent manner^15^, and mediates circadian photoreception^16^,^17^.

The zebrafish (*Danio rerio*) is an excellent circadian model and also possesses an intrinsic autonomous oscillator composed of components similar to those of mammals and flies, wherein Clock and Bmal act as positive elements and Cry and Per act as negative regulators^18^,^19^,^20^. Due to the third-round of genome duplication in vertebrate phylogeny, the teleost genome duplication (TGD), zebrafish harbors duplicates of most circadian clock genes^21^,^22^,^23^. Comparative analyses of the teleost genomes including the Japanese pufferfish (fugu) (*Takifugu rubripes*)^24^, the spotted green pufferfish (tetraodon) (*Tetraodon nigroviridis*)^25^, the Japanese medaka fish (*Oryzias latipes*)^26^, the three-spine stickleback (*Gasterosteus aculeatus*)^27^ and the zebrafish (*Danio rerio*)^28^ have revealed that zebrafish preserved *clock1a/clock1b*, *bmal1a/bmal1b* and *per1a/per1b* ancient duplicate pairs, providing insights into the evolution of teleost circadian clock genes and circadian regulatory systems^21^,^22^,^23^.

A previous study identified six zebrafish *cry* genes that were named as *zcry1a*, *zcry1b*, *zcry2a*, *zcry2b*, *zcry3* and *zcry4*^29^. Their evolutionary relationships and the mechanisms underlying their functional divergence, however, are not yet fully understood. Here, using an approach that integrates interrogation of animal genome sequences, and phylogenetic, splice site and conserved syntenic analyses, we determined that zebrafish have four *cry1* genes, *cry1aa* (*zcry1a*), *cry1ab* (*zcry1b*), *cry1ba* (*zcry2a*) and *cry1bb* (z*cry2b*); *cry2* (*zcry3*), which is the ortholog of mammalian *Cry2*; and *cry3* (*zcry4*), which is shared with amphibians, reptiles and birds but not mammals.

We observed functional divergence of these six *cry* genes, as evidenced by the sequence analyses and their diverse and rhythmic expression patterns using *in situ* hybridization and quantitative real-time PCR. Systematic cell transfection assays indicated that zebrafish possess two types of Cry proteins: Repressive Cry (RC) and Non-Repressive Cry (NRC). Zebrafish RCs, Cry1aa, Cry1ab, Cry1ba and Cry1bb, but not Zebrafish NRCs, Cry2 and Cry3, are able to repress Clock:Bmal-mediated transcription. These results shed light on the evolutionary relationships of these six zebrafish *cry* genes and lay the foundation for further elucidation of their functions.

## Results

### Evolutionary origins of zebrafish *cry* genes

**Figure 1:**
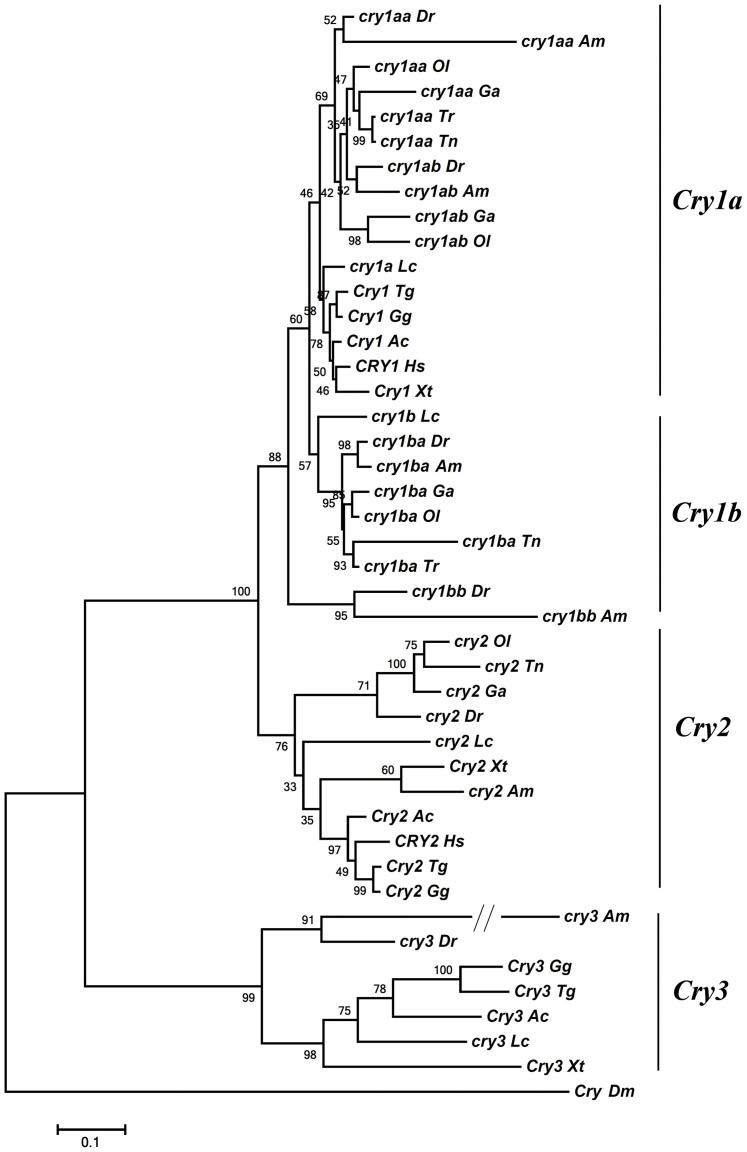
A phylogenetic tree for *Cry* genes. The tree was constructed by the Maximum Likelihood (ML) method using MEGA6^65^ with 500 bootstrap replications and the Dayhoff substitution model. Numbers on branches indicate bootstrap support values. *Dr, Danio rerio; Tr, Takifugu rubripes; Tn, Tetraodon nigroviridis; Ol, Oryzias latipes; Ga, Gasterosteus aculeatus; Am, Astyanax mexicanus; Hs, Homo sapiens; Gg, Gallus gallus; Tg, Taeniopygia guttata; Ac, Anolis carolinensis, Xt, Xenopus tropicalis*, *Lc*, *Latimeria chalumnae and Dm, Drosophila melanogaster.* The Ensembl ID numbers of these genes are listed in Supplementary Table S1.

We interrogated the six teleost fish genomes including fugu (*Takifugu rubripes*), tetraodon (*Tetraodon nigroviridis*), medaka (*Oryzias latipes*), stickleback (*Gasterosteus aculeatus*), cave fish Mexican tetra (*Astyanax mexicanus*) and zebrafish (*Danio rerio*), and several other animal genomes including of humans (*Homo sapiens*), chicken (*Gallus gallus*), zebra finches (*Taeniopygia guttata*), anoles (*Anolis carolinensis*), western clawed frogs (*Xenopus tropicalis*), coelacanths (*Latimeria chalumnae*), and fruit flies (*Drosophila melanogaster*), and uncovered a number of *Cry* genes (Supplementary Table S1). Phylogenetic analysis of these *Cry* genes showed that fish *cry1* genes (including genes previously called *zcry1a*, *zcry1b*, *zcry2a* and *zcry2b*) are all clustered with tetrapod *Cry1* genes (Fig. 1). Interestingly, these teleost *cry1* genes can be classified into two subclades, *cry1a* and *cry1b* (see more discussion below). All five teleost fishes except fugu each have one *cry2* gene, and these fish *cry2* genes (including a gene previously called *zcry3*) form a monophyletic group with tetrapod *Cry2* genes (Fig. 1). Vertebrate *Cry1* group and *Cry2* group form a sister clade, suggesting that they were derived from duplication of a common ancestral gene. In addition, in a separate monophyletic clade, zebrafish, coelacanths, frogs, anoles, zebra finches and chicken each have one *Cry3* (including a gene previously called *zcry4*) that humans lack (Fig. 1).

**Figure 2:**
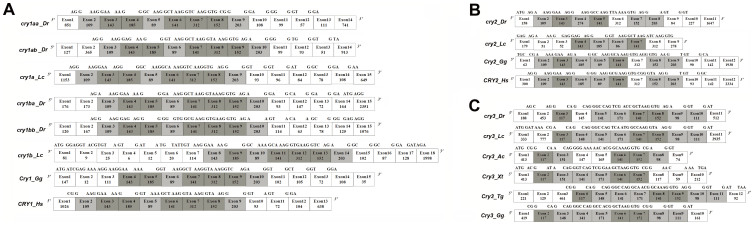
Exon/intron structures of *cry* genes. (A) Exon/intron structures of zebrafish and coelacanths *cry1a* genes, chicken *Cry1* gene and human *CRY1* gene. (B) Exon/intron structures of zebrafish and coelacanth *cry2* genes, chicken *Cry2* gene and human *CRY2* gene. (C) Exon/intron structures of zebrafish, coelacanth, frog, anole, zebra finch and chicken *Cry3* genes. The sequences at the splice sites crossing or flanking exons are shown on top of each exon boundary. *Dr, Danio rerio; Lc, Latimeria chalumnae*; *Hs, Homo sapiens; Gg, Gallus gallus; Tg, Taeniopygia guttata; Ac, Amoles carolinensis and Xt, Xenopus tropicalis*. Exon sizes are not drawn to scale. Dark grey columns indicate highly similar exons of the same size among different species and light grey columns similar exons among different species.

We also conducted splice site analysis (Figs. 2A–C and Supplementary Figs. S1A–C). Conserved exon structures including exons with the same numbers of nucleotides as well as the conserved intron phases provide evidence for gene similarities and alternative characters independent of nucleotide or amino acid sequence^21^,^22^,^23^,^30^. Most *Cry1* and *Cry2* genes except fugu *cry1aa*, tetraodon *cry1ba* and zebrafish *cry2* share linked-exons of 109 nt-143 nt-185 nt-89 nt-141 nt length at the 5′ end that all *Cry3* genes lack (Figs. 2A–C and Supplementary Figs. S1A–C), supporting the conclusion that the *Cry1* and *Cry2* clades are more closely related to each other than either is to the *Cry3* clade. In addition, most *Cry1* genes share linked-exons of 312 nt-152 nt-203 nt length, while fish *cry2* genes share linked exons of 126 nt–186 nt length (Figs. 2A and B). The relatedness of *Cry3* genes of zebrafish, coelacanths, frogs, anoles, zebra finches and chicken is supported by the observation that they all share the 117 nt exon and the four linked-exons of 141 nt-171 nt-141 nt-152 nt length (Fig. 2C). These exon structural and phylogenetic analyses of *Cry* genes generally corroborate each other (Figs. 1 and 2, Supplementary Fig. S1).

### Conserved syntenic analysis of zebrafish *cry* genes

Two or more orthologous genes linked in a single chromosome or a chromosomal fragment in each of two or more different species define a conserved syntenic region^31^,^32^. Conserved synteny analyses provide important evidence for duplication of genes and genomes. Using the BioMart function in Ensembl, we determined the orthologs or co-orthologs of five teleost fish to human genes in the chromosomal regions flanking *CRY1* or *CRY2*. The chromosomal locations and Ensembl Gene ID numbers of these genes are listed in Supplementary Table S2.

**Figure 3:**
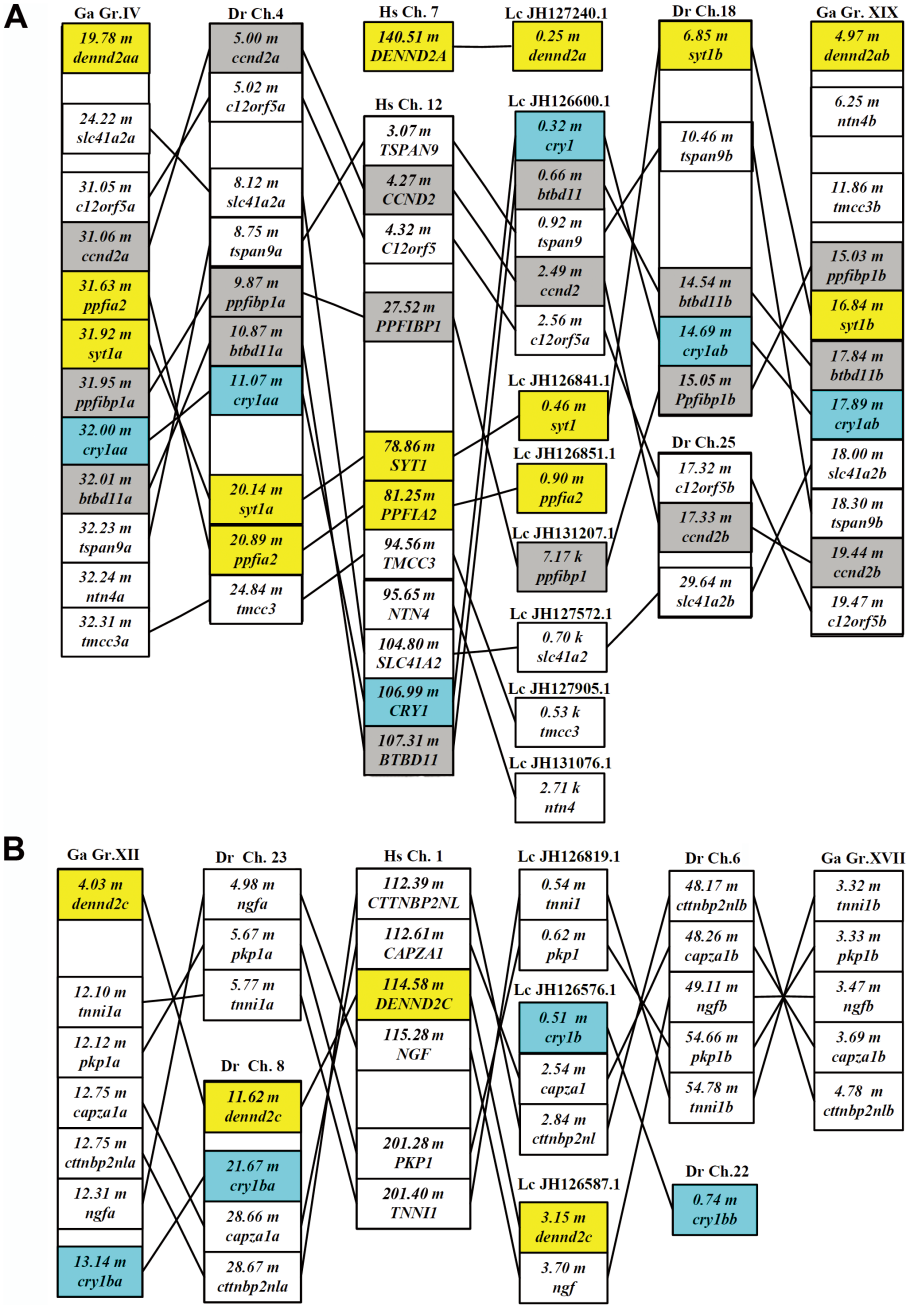
Comparison of gene orders surrounding *Cry1* in chromosomes of zebrafish, stickleback, coelacanths and human. (A) Comparison of gene orders surrounding *Cry1a* in chromosomes of zebrafish, stickleback, coelacanths and human. (B) Comparison of gene orders surrounding *Cry1b* in chromosomes of zebrafish, stickleback, coelacanths and human. Zebrafish and stickleback each have two co-orthologs for a majority of these genes flanking *CRY1* on human chromosome 12. Using stickleback *cry1ba* as an anchor site, the orthologous genes of the genes linked to *cry1b* on fish were founded in human chromosomes. The gray column indicates paralogous pairs of the human *CRY1* paralogon and the human *CRY2* paralogon. The yellow column indicates flanking paralogous pairs of *Cry1/Cry2/Cry3. Dr, Danio rerio; Ga, Gasterosteus aculeatus; Lc,Latimeria chalumnae; Hs, Homo sapiens.* The Ensembl ID numbers of these genes are listed in Supplementary Table S2. Abbreviations: m, million base pairs from one end of the chromosome, linkage group, or scaffold where the gene is located. The positions of genes on chromosomes are not drawn to scale.

Zebrafish, medaka and stickleback each have two co-orthologs for a majority of these genes flanking *CRY1* on human chromosome 12 (Fig. 3A and detailed result in Supplementary Fig. S2A), for instance, zebrafish have two co-orthologs for *C12orf5*, *CCND2, BTBD11, PPFIBP1* and *TSPAN9* that are linked to *CRY1* in human chromosome 12 (Fig. 3A and Supplementary Fig. S2A). Moreover, zebrafish co-orthologs for these genes flanking *CRY1* on human chromosome 12 are located in two different zebrafish chromosomes, for instance, zebrafish *btbd11a* is located in chromosome 4, while *btbd11b* is located in chromosome 18 (Fig. 3 and Supplementary Fig. S2). Zebrafish *ccnd2a* and *c12orf5a* are located in the paralogon defined by *cry1aa*, *tspan9a, ppfibp1a* and *btbd11a*, whereas *ccnd2b* and *c12orf5b* are likely translocated to chromosome 25 from chromosome 18 after the duplication (Fig. 3A and Supplementary Fig. S2A). Interestingly, we observed that a synteny defined by *Cry1b*, *Capza1* and *Cttnbp2nl* is conserved between coelacanths (JH126576.1) and tetrapods including humans (chromosome 1) and chicken (chromosome 26), even though tetrapods had lost the putative *Cry1b* gene (Fig. 3B and Supplementary Fig.S2B). This *cry1b*-*capza1*-*cttnbp2nl* synteny corresponds to a pair of paralogons in teleost fishes, for instance, *cry1ba*, *capza1a*, *cttnbp2nla* on stickleback linkage group XII and zebrafish chromosome 8, whereas stickleback *capza1b* and *cttnbp2nlb* are linked on linkage group XVII in which the putative *cry1bb* was lost, and zebrafish *capza1b* and *cttnbp2nlb* are linked on chromosome 6 but *cry1bb* is on chromosome 22 (Fig. 3B and Supplementary Fig.S2B). Thus, it appears that the part of human chromosome 12 harboring *CRY1* (or called *CRY1A*, see discussion below) corresponds to a pair of teleost fish chromosomes or linkage groups harboring *cry1aa/cry1ab* (Fig. 3 and Supplementary Fig. S2), and the part of human chromosome 1 in which the putative *CRY1B* was lost corresponds to a pair of teleost fish chromosomes or linkage groups harboring *cry1ba* and *cry1bb* (Fig. 3 and Supplementary Fig. S2).

**Figure 4:**
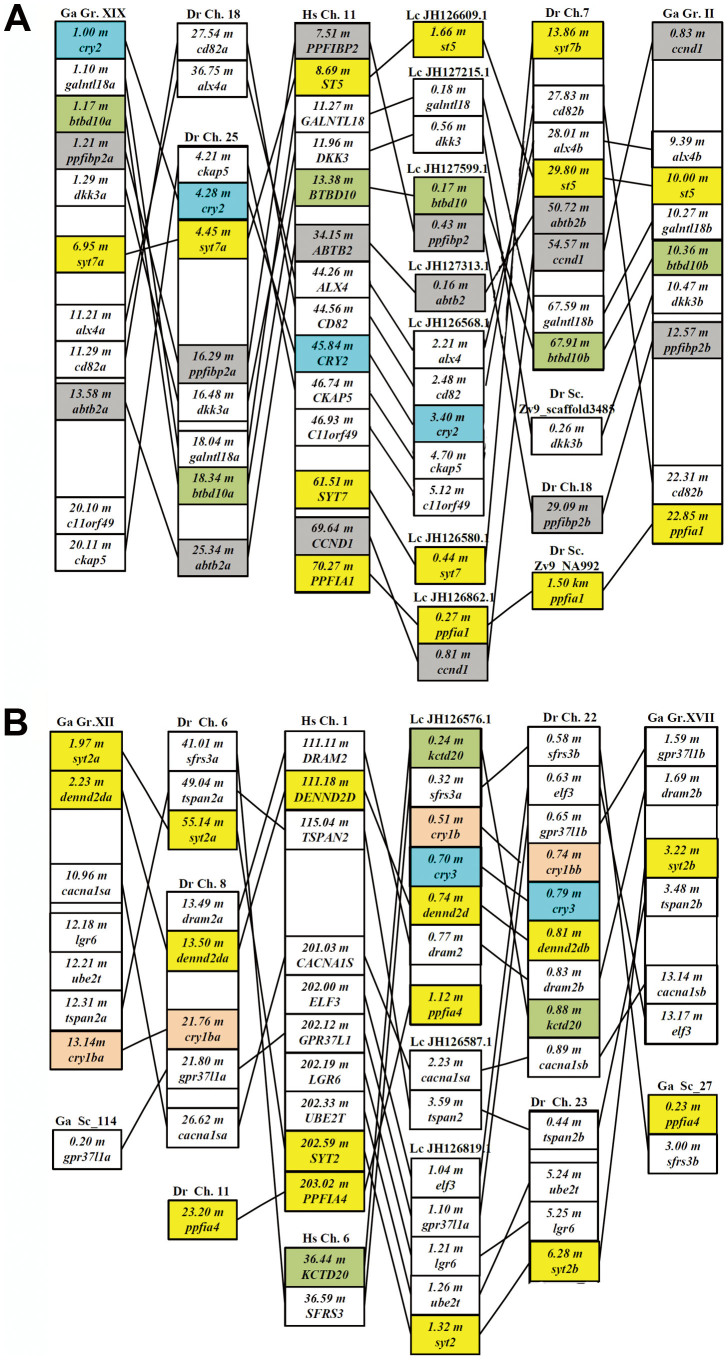
Comparison of gene orders surrounding *Cry2* or *Cry3* genes. (A) Comparison of gene orders surrounding *CRY2* in chromosomes of zebrafish, stickleback, coelacanths and human. The human *CRY2* region corresponds to two fish paralogons; one paralogon contains *cry2a*, the other paralogon contains some co-orthologs for genes flanking *CRY2* on human chromosome 11 but the putative *cry2b* was lost. (B) Comparison of gene orders surrounding *cry3* and *cry1b* in chromosomes of zebrafish, stickleback, coelacanths and human. Gray boxes indicate paralogous pairs of the human *CRY1* paralogon and the human *CRY2* paralogon. Yellow boxes indicate paralogous pairs of flanking genes. *Dr, Danio rerio; Ga, Gasterosteus aculeatus; Lc, Latimeria chalumnae; Hs, Homo sapiens.* Ensembl ID numbers of these genes are listed in Supplementary Table 2. Abbreviations: m, million base pairs from one end of the chromosome, linkage group, or scaffold where the gene is located. Positions of genes on chromosomes are not drawn to scale.

Conserved synteny analysis showed that teleost genome duplication (TGD) also generated two *cry2* genes but only one, the ortholog of tetrapod *Cry2* (Figs. 1 and 4A, Supplementary Fig. S3A), was retained in the fish lineage (Fig. 4A and Supplementary Fig. S3A). Human *CRY2* is closely linked to *PPFIBP2*, *GALNTL18*, *DKK3*, *BTBD10*, and *ALX4* on human chromosome 11 (Fig. 4A and Supplementary Fig.S3A). We observed that two paralogons of this fragment of human chromosome 11 arose in the teleost genome duplication (TGD), however, only one paralogon (for instance, on zebrafish chromosome 25 and stickleback linkage group XIX) retains a *cry2* gene in the fish lineage, the other paralogon still exists but lost its *cry2* gene following the TGD (Fig. 4A and Supplementary Fig. S3A). Moreover, it is noteworthy that *cry2* and *cry1ab* were found in the same chromosome/linkage group in medaka (Ol chromosome 6) and stickleback (Ga Group XIX); and in zebrafish, the two *cry* genes also appear to be in the same chromosome before, and now are distributed in chromosomes 25 and 18, respectively (Figs. 3A and 4A and Supplementary Figs. S2A and S3A).

Although humans do not possess *CRY3* gene (Fig. 1), we still observed a highly conserved synteny of the chromosome segment that originally contained this gene among lobe-finned fish including human chromosome 1, chicken chromosome 26, anole chromosome 10 and coelacanth JH1265576.1, defined by *Cacna1s*, *Dennd2d*, *Dram2*, *Gpr37l1* and *Tspan2* (Fig. 4B and Supplementary Fig. S3B). Likewise, the TGD generated two orthologs of these genes and several others distributed in teleost fish chromosomes and linkage groups (Fig. 4B and Supplementary Fig. S3B). Intriguingly, coelacanth *cry3* and *cry1b*, and zebrafish *cry3* and *cry1bb*, were found to be closely linked together (Fig. 4B and Supplementary Fig. S3B), strongly suggesting an origin of *cry3* and *cry1b* (or *cry1bb*) by local (or tandem) duplication. Furthermore, the observation that ray-finned fish (zebrafish) and lobe-finned fish (coelacanths) share such a close linkage of *cry3*-*cry1b* would implicate that the local (or tandem) duplication must have been in place before the divergence of bony vertebrates, likely before the first round of vertebrate genome duplication (VGD). Even though frogs, anoles and chicken each maintained one *Cry3* located solely in a chromosome, and one *Cry1* (likely *Cry1a*) in another chromosome, all the three lineages had lost the putative *Cry1b* gene and the *Cry3-Cry1b* linkage (Fig. 4B and Supplementary Fig. S3B). In teleost fish, due to differential gene losses after the TGD, while zebrafish retained the *cry1ba/cry1bb* pair, one *cry3* and the *cry3-cry1bb* linkage, fugu, medaka, stickleback and tetraodon retained only one *cry1ba* but lost *cry3* and the *cry3-cry1bb* linkage (Fig. 1, Fig. 4B and Supplementary Fig. S3B).

To verify our syntenic analyses, we also used synteny database (http://syntenydb.uoregon.edu/synteny_db/)^33^. By comparing stickleback with humans, we found the conserved syntenic regions defined by the same sets of genes for *CRY1* (Supplementary Fig. S4A) and *CRY2* (Supplementary Fig. S4B), thereby supporting validity of our conserved syntenic analysis.

### Function divergence analysis of zebrafish *cry* duplicates

Numerous studies have addressed the underlying mechanisms of functional divergence after duplication^34^,^35^,^36^,^37^. Type I function divergence in a gene family refers to amino acid configurations that are highly conserved in gene 1 but highly variable in gene 2, or *vice versa*, in a gene family, which is estimated by the coefficient of functional divergence (θ)^35^. Previous studies indicated that significant type I function divergence after gene duplication leads to altered selective constraints and functional specification between duplicate genes^35^,^38^,^39^,^40^,^41^. By comparing Cry1and Cry2/Cry3, and Cry1/Cry2, we found that Cry proteins show statistically significant altered selective constraint and functional specification in comparisons of Cry1 and Cry2/Cry3, and Cry1/Cry2 (p < 0.01) (Table 1). The selective constraints in the pairs of Cry1a/Cry1b, Cry1a/CRY2 and Cry1a/CRY3 also were significantly altered (p < 0.01) (Table 1). A previous study investigated protein structure and function for zebrafish Cry1aa (Cry1a in the old nomenclature) protein structure and function, and validated that RD-2a (aa126–196) or RD-1 (aa197–263) regions in the peptide chain are required for interaction with the Clock: Bmal heterodimer, while RD-1 or RD-2b (aa264–293) regions are required for nuclear translocation of Cry1aa protein^42^. Both of these two functional domains are required for the transcriptional repression activity of zebrafish Cry1aa. Therefore, the critical amino acid residues that are responsible for the functional divergence were revealed by the amino acid sequence of zebrafish Cry1aa, consistent with our Type I function divergence analyses (Table 2). In addition to Cry1a/Cry1b, numerous sites with the posterior probability Qk > 0.67 among a total of 327 sites in pairs studied here were predicted to be responsible for type I functional divergence^35^,^38^ (Tables 1 and 2). For example, in the Cry1a/Cry2 pair, 18 of 28 sites with the posterior probability Qk > 0.67 in the region are required for interaction with the Clock: Bmal1 heterodimer, while in comparison with Cry1a, Cry2 and Cry3 have different numbers of the sites in the RD-2b region required for nuclear translocation (Table 2).

We also conducted Tajima relative rate tests^43^ to investigate whether one of the fish gene duplicates for the *cry1aa/cry1ab* or *cry1ba/cry1bb* pairs had evolved at an accelerated rate following the teleost genome duplication. A statistically significant increase in evolutionary rate was found in one of the duplicates for zebrafish *cry1aa/cry1bb* but not for zebrafish *cry1aa/cry1ab* (Supplementary Table S3). These molecular evolutionary analyses suggest that zebrafish *cry* genes have evolved divergent functions.

### Diverse and rhythmic expression patterns of zebrafish *cry* genes

**Figure 5:**
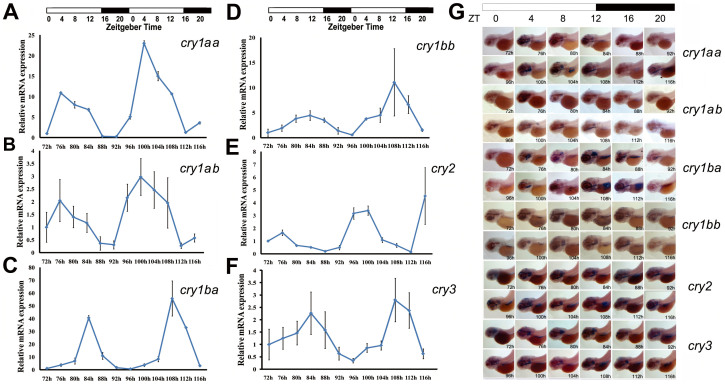
Diverse and rhythmic expression patterns of zebrafish *cry* genes. (A) Rhythmic expression of zebrafish *cry1aa* determined by qRT-PCR. (B) Rhythmic expression of zebrafish *cry1ab* determined by qRT-PCR. (C) Rhythmic expression of zebrafish *cry1ba* determined by qRT-PCR. (D) Rhythmic expression of zebrafish *cry1bb* determined by qRT-PCR. (E) Rhythmic expression of zebrafish *cry2* determined by qRT-PCR. (F) Rhythmic expression of zebrafish *cry3* determined by qRT-PCR. Each value is the mean ± SD of the three independent experiments. (G) Lateral views (anterior to left) of expression patterns of zebrafish *cry1aa, cry1ab, cry1ba, cry1bb, cry2* and *cry3* shown by *in situ* hybridization. Whole-mount *in situ* hybridization was performed to examine expression patterns of six *cry* genes from 72 hpf to 116 hpf (Day 4 and Day 5 post fertilization, at 4 h intervals each day). Black in the horizontal bars at the top represent darkness, and white bars indicate light. Zebrafish *cry1aa, cry1ab, cry1ba, cry1bb, cry2* and *cry3* display diverse patterns of rhythmic expression in larvae. *cry1aa* and *cry1ab* display distinct but similar rhythmic expression patterns, both reaching a trough at ZT15-19. The oscillating patterns of *cry1ba* and *cry1bb* are similar, both peaking at ZT13-15. The oscillating patterns of *cry2* and *cry3* differed from each other: *cry2* peaked at ZT23-1, while *cry3* peaked at ZT9. *In situ* hybridization results are consistent with those of qRT-PCR.

To further examine functional divergence of these six zebrafish *cry* genes, we used both quantitative real-time PCR (qRT-PCR) and whole-mount *in situ* hybridization (WISH) to examine their expression patterns. A total of 12 larval stages from 72 hpf (hours post fertilization) to 116 hpf over two consecutive days (4-hour intervals, 6 stages each day, approximately 30 larvae per sample) under LD (light/dark) condition were examined. Results clearly demonstrated that all six zebrafish *cry* genes are rhythmically expressed in a distinct and robust manner (Fig. 5). Both *cry1aa* and *cry1ab* displayed similar oscillating expression patterns: both peaked at noon (Zeitgeber time, ZT4) and reached a trough at dawn (ZT16-20)(Figs. 5A and B). The oscillating patterns of *cry1ba* and *cry1bb* differ from those of *cry1aa* and *cry1ab* in that *cry1ba* and *cry1bb* both peaked at the night (ZT12) and reached the nadir in the morning (ZT0) instead (Figs. 5C and D). Both *cry2* and *cry3* were also expressed rhythmically: *cry*2 peaked at noon (ZT4), while *cry*3 peaked at night (ZT12) (Figs. 5E and F). qRT-PCR results were consistent with our *in situ* hybridization analysis (Fig. 5G, Supplementary Fig. S5). Importantly, our *in situ* hybridization results clearly showed that these *cry* genes oscillated in the eye, the liver or the ear (Fig. 5G and Supplementary Fig. S5). Both qRT-PCR and *in situ* hybridization analyses corroborated the functional divergence revealed by Type I function divergence analyses and Tajima relative rate tests discussed above.

### Repression of zebrafish Cry proteins on Clock:Bmal-mediated transcription

**Figure 6:**
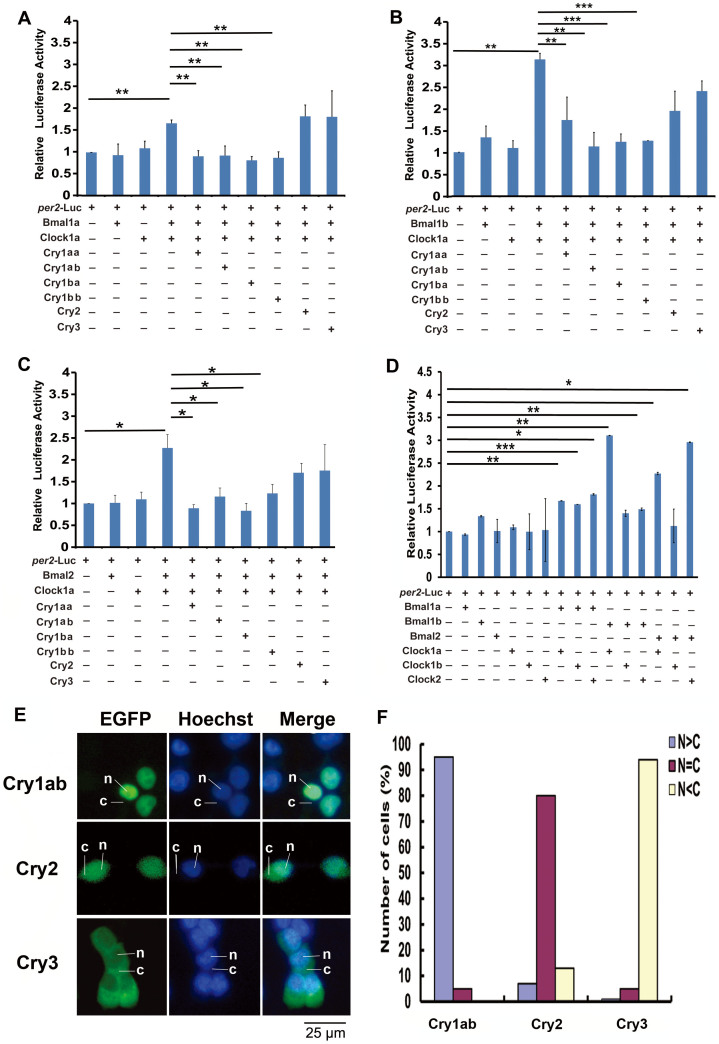
Repression of zebrafish Cry proteins on different Clock:Bmal combinations. (A) Clock1a:Bmal1a combination, (B) Clock1a:Bmal1b combination and (C) Clock1a:Bmal2 combination. (D) All possible Clock:Bmal combinations. Each value is the mean ± SD of the three independent experiments. Results were analyzed by ANOVA. One star on the line indicates 0.01 < p < 0.05, two stars on the line p < 0.01, and three stars on the line p < 0.001. (E) Sub-cellular localizations of zebrafish Cry1ab, Cry2 and Cry3. Cry1ab-EGFP, Cry2-EGFP or Cry3-EGFP was transfected into HEK293 cells, respectively, and then the cells were stained with Hoechst and observed by an inverted fluorescent microscopy. The cytoplasmic or nuclear distribution of zebrafish Cry1ab, Cry2 or Cry3 was detected by GFP signal (green); DNA was stained by Hoechst (blue), respectively. The leader lines and letters N, C indicate the location of nucleus and cytoplasm, respectively. (F) Quantitative analysis of the subcellular localization of each Cry proteins. In each experiment, 50–100 cells were evaluated for nuclear fluorescence (N > C, blue bars), nuclear-cytoplasmic fluorescence (N = C, purple bars), and cytoplasmic fluorescence (N < C, pink bars). The figure shows a representative of the three independent experiments.

The current model for zebrafish circadian rhythmicity is an autoregulatory feedback loop in which heterodimers formed by Clock and Bmal proteins activate transcription of *per* and *cry* genes by binding the E/E′ boxes in *per* and *cry* gene regulatory regions, and Per and Cry proteins form heterodimers that enter the nucleus, bind to Clock:Bmal heterodimers and inhibit transcription^18^,^19^,^20^. To systematically examine effects of these six Cry proteins on all nine possible Clock:Bmal combinations, we used a 450 bp long promoter fragment from the zebrafish core clock gene *per*2, which carries one E box and three E′ boxes^69^, and the full length cDNAs of all three zebrafish *clock* genes^22^,^44^, three *bmal* genes^21^,^44^ and six *cry* genes^29^, to conduct the dual luciferase reporter gene assays with HEK 293 cells. Luciferase assays showed that Cry1aa, Cry1ab, Cry1ba and Cry1bb inhibit Clock:Bmal-mediated transcription, whereas Cry2 and Cry3 do not. Thus, it appears that zebrafish possesses two types of Cry proteins, Repressive Cry (RC) including Cry1aa, Cry1ab, Cry1ba and Cry1bb and Non-Repressive Cry (NRC) including Cry2 and Cry3, consistent with a previous observation^29^. Our results showed that except for the Clock1b:Bmal2 and Clock1b:Bmal1b combinations, all other Clock:Bmal heterodimer combinations showed statistically significant transactivation activities, although various combinations showed differences in transactivating efficiencies (Figs. 6A–C, Supplementary Figs. S6A–F). Among interactions, the Clock1b:Bmal1a combination had the lowest transactivating efficiency (Fig. 6D). Sequence alignments showed that Clock1b lacks of one typical protein-interaction PAS domain (data not shown), which probably limits its transactivation ability.

### Subcellular localizations of Cry1ab, Cry2 and Cry3 and the novel Cry 1bb nuclear localization signal (NLS) sequence

Although the six zebrafish Cry proteins share similar amino acid sequences, the effects they exert on Clock:Bmal-mediated transcription are different. Our functional divergence analysis showed that zebrafish Cry2 and Cry3 differ substantially from Cry1aa in amino acid sequences of both the protein interaction domain and the nuclear translocation domain (Table 2). We used EGFP-fused protein expression experiments to examine subcellular localizations of Cry1ab, Cry2 and Cry3. Results showed that Cry1ab, which effectively represses the Clock:Bmal-mediated transcription, displayed predominantly nuclear localization (Figs. 6E and F). In contrast, the two non-repression Cry proteins failed to accumulate in the nucleus: Cry3 predominantly located in the cytoplasm, whereas Cry2 distributed throughout the cytoplasm and nucleus (Fig. 6E and F).

Nuclear localization signal (NLS) sequences of Cry proteins are important for their ability to enter the nucleus and acting as inhibitors^45^. We used the program NLStradamus (www.moseslab.csb.utoronto.ca/NLStradamus)^46^ to examine whether zebrafish Cry proteins have NLS sequences. Results showed that except for Cry3, all other Cry proteins, Cry1aa, Cry 1ab, Cry 1ba, Cry 1bb and Cry 2 possess predicted NLS sequences (Supplementary Fig. S7). Moreover, the predicted NLS sequences of Cry1aa, Cry 1ab, and Cry 1ba are located in the middle of their protein sequences, while the NLS sequence of Cry 1bb is at its C-terminus (Cry1ba also has a second predicted NLS at its C-terminus) (Supplementary Fig. S7).

**Figure 7:**
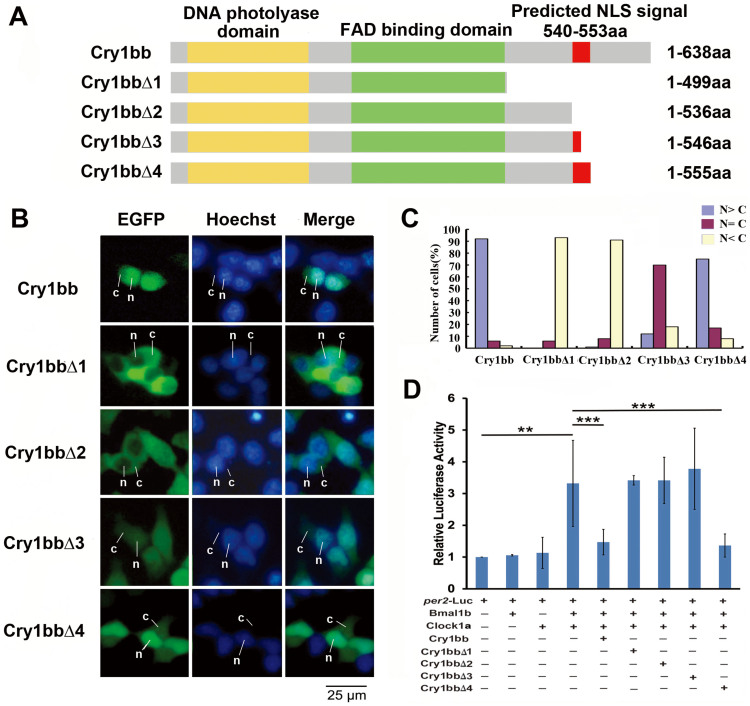
Identification of a novel NLS (Nuclear Localization Signal) sequence of zebrafish Cry1bb. (A) Schematic diagrams of the full length of zebrafish Cry1bb (1–638aa) and Cry1bb truncation constructs. The yellow bar indicates the DNA photolyase domain, the green bar indicates the FAD binding domain of Cry proteins and the red bar indicates the predicted NLS sequence (VRREQPGPSGAKHR, Ensembl Peptide ID: ENSDARP00000090995) according to an online program (www.moseslab.csb.utoronto.ca/NLStradamus)^46^ (see also Supplementary Fig. S7). Cry1bbΔ1 contained 499 amino acids, truncated at amino acid position number 499; Cry1bbΔ2 contained 536 amino acids truncated at amino acid position number 536 just before the predicted NLS signal; Cry1bbΔ3 contained 546 amino acids truncated at amino acid position number 546 in the middle of the predicted NLS signal; and Cry1bbΔ4 contained 555 amino acids truncated at amino acid position number 555 just after the predicted NLS signal. (B) Sub-cellular localization of zebrafish Cry1bb and its four truncated fragments. Cry1bb-EGFP or Cry1bb-EGFP truncated fragments were transfected into HEK293 cells, respectively, and then the cells were stained with Hoechst. The cytoplasmic or nuclear distributions of zebrafish Cry1bb and its four truncated fragments were detected by GFP signals (green); the nuclei were identified by Hoechst (blue). The leader lines and letters N, C indicate nuclear and cytoplasmic localizations, respectively. (C) Quantitative analysis of the subcellular localization of each Cry proteins. In each experiment, 50–100 cells were evaluated for nuclear fluorescence (N > C, blue bars), nuclear-cytoplasmic fluorescence (N = C, purple bars), and cytoplasmic fluorescence (N < C, pink bars). The figure shows a representative of the three independent experiments. (D) Repressive activities of zebrafish Cry1bb and its four truncated fragments determined by dual Luciferase reporter assays. The full length of Cry1bb and its four truncated fragments were co-transfected with *per2*-luc reporter and Bmal1b:Clock1a combination, respectively. Among the four Cry1bb truncated fragments, only Cry1bbΔ4 retained the repressive ability of the full length Cry1bb on Bmal1b:Clock1a-mediated transcription. A Renilla Luciferase was added in each transfection to normalize transfection efficiency. The figure shows the mean and SD (error bar) of two independent experiments (triplicate for each experiment). Results were analyzed by ANOVA.

To examine whether the predicted Cry 1bb NLS sequence functions properly, we generated four EGFP-fused proteins with truncated fragments of Cry1bb based on the predicted NLS: Cry1bbΔ1 contained 499 amino acids, truncated at amino acid position number 499; Cry1bbΔ2 contained 536 amino acids, truncated at amino acid position number 536, right before the predicted NLS signal (aa540-aa553); Cry1bbΔ3 contained 546 amino acids, truncated at amino acid position number 546, in the middle of the predicted NLS signal (aa540-aa553); and Cry1bbΔ4 contained 555 amino acids, truncated at amino acid position number 555, just after the predicted NLS signal (aa540–aa553) (Fig. 7A). We examined subcellular localizations of these four Cry1bb truncated EGFP-fusion proteins. As expected, full-length Cry1bb showed predominant nuclear localization. In contrast, the three Cry1bb truncation proteins, Cry1bbΔ1 and Cry1bbΔ2 that both lacked the predicted NLS sequence, and Cry1bbΔ3 that had only part of the predicted NLS, were located primarily in the cytoplasm. Only Cry1bbΔ4, which contained the predicted NLS sequence was translocated into the nucleus (Fig. 7C). We also conducted cell transfection assays to examined inhibitory abilities of these four truncated Cry1bb proteins on Clock:Bmal-mediated transcription. Results showed that only Cry1bbΔ4, which we showed to enter the nucleus, repressed Clock1a:Bmal1b-mediated activation, while the other three truncated proteins Cry1bbΔ1, Cry1bbΔ2 and Cry1bbΔ3, which were located primarily in the cytoplasm, failed to repress Clock1a:Bmal1b-mediated activation (Fig. 7D). These results confirmed that the predicted NLS of Cry1bb in the C-terminus is functional, which differs from the predicted NLS located in the middle of the protein sequence that was identified previously for zebrafish Cry1aa^45^.

### Mechanistic explanations for non-repression of zebrafish Cry2 and Cry3

**Figure 8:**
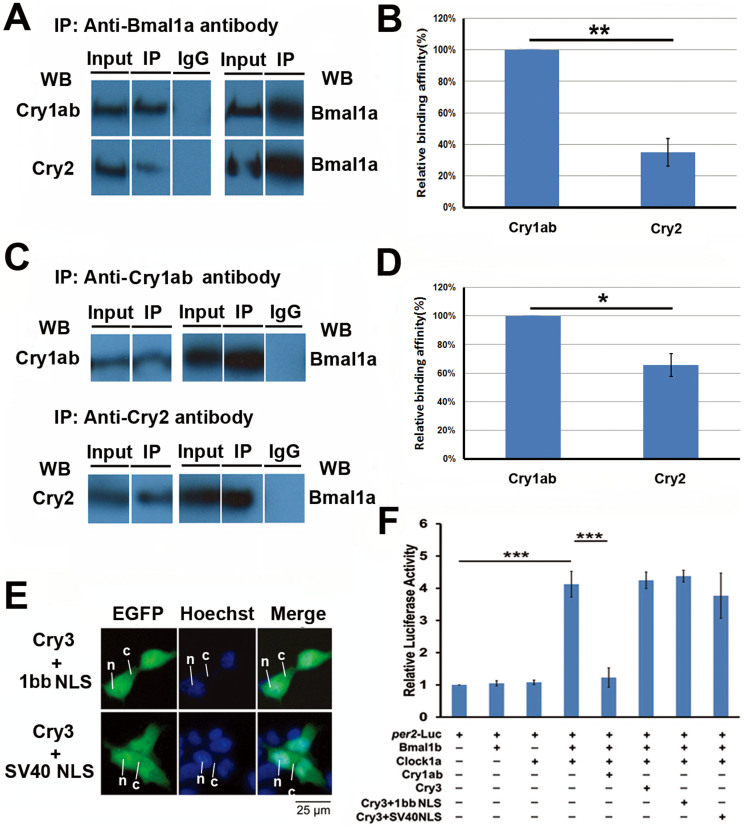
Mechanisms for non-repression of zebrafish Cry2 and Cry3 on Clock:Bmal mediated transcription. (A) Co-immunoprecipitation (Co-IP) experiments. Zebrafish *cry1ab* and *cry2* constructs were co-transfected into HEK293 cells with *per2*-Luc reporter and *bmal1a*, and *clock1a*, respectively. Then the anti-Bmal1a polyclonal antibody was used to pull down proteins bound to Bmal1a, which was identified by the anti-Cry1ab and anti-Cry2 polyclonal antibodies, respectively. (B) The relative protein binding affinity of Cry1ab and Cry2 to Bmal1a-Clock1a complex. The relative protein binding affinity was quantified with Image pro-Plus6.0. Each value is the mean ± SD of three independent experiments. Results were analyzed by Student's *t*-test. Horizontal lines indicate columns compared for significance. One star on the line shows 0.01 < p < 0.05. (C) Protein binding affinity comparison of Cry1ab and Cry2 to Bmal1a:Clock1a complex. Zebrafish *cry1ab* and *cry2* constructs were co-transfected into HEK293 cells with *per2*-luc reporter and Bmal1a-Clock1a combination, respectively. Then anti-Cry1ab or anti-Cry2 polyclonal antibody was used to collect the proteins bound to Bmal1a, which was identified by the anti-Bmal1a polyclonal antibody. (D) The relative protein binding affinity of Cry1ab and Cry2 to the Clock1a-Bmal1a complex from Fig 8*C*. The relative protein binding affinity was quantified by Image pro-Plus6.0. Each value is the mean ± SD of three independent experiments. Results were analyzed by Student's *t*-test. Horizontal lines indicate columns compared for significance. Two stars on the line means p < 0.01, (E) Sub-cellular localization of zebrafish Cry3 fused to either Cry1bb NLS from Fig 7A or a SV40 NLS. The Cytoplasmic-nuclear distribution of these two DNA constructs was detected by GFP signals (green) and the nucleus identified by Hoechst (blue). The leader lines and letters N, C indicate the location of nucleus and cytoplasm, respectively. (F) Inhibitory activities of zebrafish Cry3 fused to either Cry1bb NLS or a SV40 NLS determined by dual Luciferase reporter assays. The two DNA constructs were co-transfected with *per2*-Luc reporter and a Clock1a-Bmal1b combination, respectively. A Renilla Luciferase was added in each transfection to normalize transfection efficiency. The figure shows the mean and SD (error bar) of the two independent experiments (triplicate for each experiment). Results were analyzed by ANOVA.

We then examined hypotheses for the mechanistic explanations for non-repression of zebrafish Cry2 and Cry3. In contrast to Cry3, zebrafish Cry2 was able to enter the nucleus to a certain degree, so why does it fail to inhibit Clock:Bmal-mediated transcription? Because Cry2 differs from Cry1aa in the protein interaction domain (RD-2a and RD-1) based upon our type I function divergence analysis (Table 2), we assumed that zebrafish Cry2 might fail to bind to the Clock:Bmal heterodimer *in vivo* and thus subsequently fail to repress Clock:Bmal-mediated transcription. We carried out co-immunoprecipitation experiments to examine this hypothesis. Results showed that while Cry1ab directly bound to Bmal1a, Cry2 was only weakly co-precipitated with Bmal1a (Figs. 8A–D), suggesting that non-repression of Cry2 is due to its non-effective binding to the Clock:Bmal heterodimer.

Zebrafish Cry3 cannot enter the nucleus, and thus cannot repress Clock:Bmal-mediated transcription. Does Cry3 inhibit Clock:Bmal-mediated transcription if it enters the nucleus? To examine this possibility, we generated two chimeric proteins that fused Cry3 to either the Cry1bb NLS sequence we just identified or to the SV40 NLS sequence^47^, respectively. Results indicated that both NLS sequences can translocate Cry3 into the nucleus (Fig. 8E); nevertheless, even when it was inside the nucleus, Cry3 still failed to inhibit Clock:Bmal-mediated transcription (Fig. 8F). It appears that zebrafish Cry3 has evolved neither a protein-protein interaction domain nor an NLS sequence, both of which are required for transcription repression activity.

## Discussion

### Evolution of zebrafish *cry* genes

**Figure 9:**
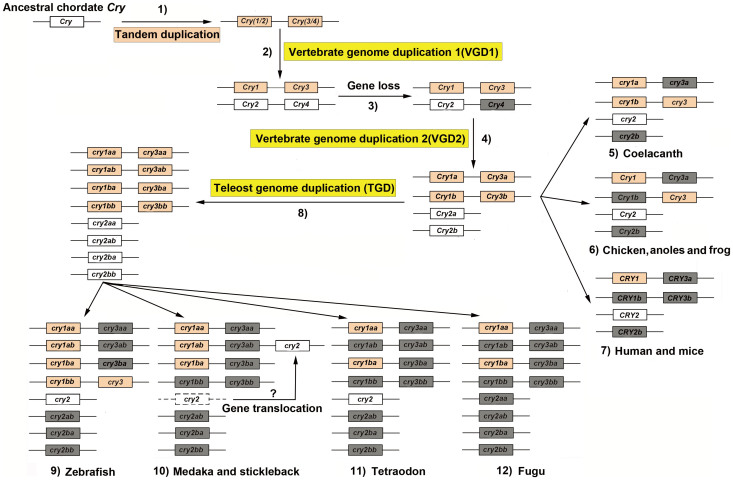
A hypothetical model for evolution of the teleost fish *cry* genes. The most likely scenario of *Cry* evolution is proposed below: *Cry* genes of teleost fish and tetrapods have a common ancestor; 1) a local (or tandem) duplication of the chordate ancestral *Cry* gene occurred likely before the first round of vertebrate genome duplication (VGD), and gave rise to *Cry12* and *Cry34*; 2) during VGD1, *Cry12* gave rise to *Cry1* and *Cry2*, while *Cry34* gave rise *Cry3* and *Cry4* with the *Cry1-Cry3* and *Cry 2-Cry4* close linkages; 3) *Cry4* was subsequently lost; 4) during the second round of VGD, *Cry1*, *Cry2*, and *Cry3* were duplicated to generate *Cry1a/Cry1b*, *Cry2a/Cry2b* and *Cry3a/Cry3b*; 5) due to differential gene losses after the second VGD, coelacanths retained *cry1a*, *cry1b*, *cry2*, *cry3* and the *cry3-cry1b* close linkage (in brown); 6) chicken, anoles and frogs retained *Cry1*(*Cry1a*), *Cry2* and *Cry3*; 7) humans and mice retained *Cry1*(*Cry1a*) and *Cry2*; 8) during the teleost genome duplication (TGD), teleost fish *cry* genes were duplicated; 9) following the TGD and subsequent gene losses, zebrafish retained *cry1aa/cry1ab*, *cry1ba/cry1bb*, *cry2* and *cry3* and the *cry3-cry1bb* linkage (in brown); 10) medaka and stickleback retained *cry1aa/cry1ab*, *cry1ba* and *cry2*; 11) tetraodon retained *cry1aa, cry1ba* and *cry2*; and 12) fugu preserved *cry1aa* and *cry1ba*. A translocation event occurred likely in teleost fishes before their radiation following the TGD generated the *cry2-cry1ab* synteny that was preserved in medaka and stickleback. Genes in the grey boxes were lost during evolution.

Our phylogenetic, splice site and syntenic analyses support the notion that *Cry* genes in the teleost fish and tetrapods share a common ancestor (Fig. 9): 1) a local (or tandem) duplication of chordate ancestral *Cry* occurred likely before the first round of vertebrate genome duplication (VGD), and gave rise to *Cry12* and *Cry34*; 2) during the first round of VGD, *Cry12* gave rise to *Cry1* and *Cry2*, while *Cry34* gave rise *Cry3* and *Cry4* with *Cry1* as the immediate neighbor of *Cry3* and *Cry2* as the immediate neighbor of *Cry4*; 3) *Cry4* was subsequently lost; 4) during the second round of VGD, *Cry1*, *Cry2*, and *Cry3* were duplicated to generate *Cry1a/Cry1b*, *Cry2a/Cry2b* and *Cry3a/Cry3b*; 5) due to differential gene losses after the second VGD, coelacanths retained *cry1a*, *cry1b*, *cry2* and *cry3* and the *cry3-cry1b* close linkage; 6) chicken, anoles and frogs retained *Cry1* (*Cry1a*), *Cry2* and *Cry3*; 7) humans and mice retained only *Cry1* (*Cry1a*) and *Cry2*; 8) during the third round of teleost genome duplication (TGD), teleost fish *cry1a*, *cry1b*, *cry2* and *cry3* were duplicated, respectively; 9) following the TGD and subsequent gene losses, zebrafish retained *cry1aa/cry1ab*, *cry1ba/cry1bb*, *cry2*, *cry3* and the *cry3-cry1bb* linkage; 10) medaka and stickleback retained *cry1aa/cry1ab*, *cry1ba* and *cry2*; 11) tetraodon retained *cry1aa, cry1ba* and *cry2*; and 12) fugu preserved *cry1aa* and *cry1ba* (Fig. 9).

We propound that a local (or tandem) duplication of chordate ancestral *Cry* occurred likely before the first round of vertebrate genome duplication (VGD) based on following lines of evidence: (1) The phylogenetic and splice site analyses showed that fish *cry1a* resembles *cry1b* more than *cry2* and *cry3* (Figs. 1 and 2); and the type I functional divergence analyses also showed that Cry1a/Cry1b had fewer number of sites with the posterior probability Qk > 0.67 than other five pairs compared (Table 1). These results indicated that *cry1a* and *cry1b* are more closely related evolutionarily to each other than to any of the other *Cry* genes; (2) Conserved syntenic analysis showed that the part of human chromosome 12 harboring *CRY1* (or called *CRY1A*) corresponds to a pair of teleost fish chromosomes or linkage groups harboring *cry1aa/cry1ab* (Figs. 3 and Supplementary Fig. S2), and the part of human chromosome 1 which would have formerly contained the putative *CRY1B* corresponds to a pair of teleost fish chromosomes or linkage groups harboring *cry1ba/cry1bb* (Figs. 3 and Supplementary Fig. S2); and (3) importantly, in lobe-finned coelacanth, which did not undergo the TGD^48^, *cry3* and *cry1b* are closely linked in JH1265576.1 (Fig. 4B, Supplementary Fig. S3B), and in ray-finned zebrafish that did have the TGD, *cry3* and *cry1bb* are closely linked in chromosome 22 (Fig. 4B and Supplementary Fig. S3B), supporting that the local (tandem) duplication that generated the *cry3-cry1b* close linkage occurred likely before the first VGD. In contrast, because the *cry2-cry1ab* synteny with a relatively long distance was preserved only in medaka and stickleback (Figs. 3A and 4A, Supplementary Figs. S2A and S3A), we posit that a translocation event occurred likely in teleost fishes before their radiation following TGD generated it (Fig. 9). Our previous evolutionary analyses of teleost fish *clock*, *bmal* and *period* revealed that extra duplicate pairs of circadian clock genes were consistent with the VGD and TGD hypotheses, the most parsimonious evolutionary trajectory for occurrences of these extra copies^21^,^22^,^23^. Similarly, the current study of teleost fish *cry* genes also supports the VGD and TGD hypotheses for generation of additional duplicate pairs of *cry1aa*/*cry1ab* and *cry1ba*/*cry1bb* (Fig. 9).

These six zebrafish *cry* genes were previously called *zcry1a*, *zcry1b, zcry2a, zcry2b, zcry3* and *zcry4*^29^. Based upon our comparative genomic analysis, these genes should be renamed as *cry1aa*, *cry1ab, cry1ba, cry1bb, cry2 and cry3*, respectively, to better indicate their evolutionary relationships and increase genome connectivity.

### Functional divergence of zebrafish *cry* genes

Subfunctionalization^49^,^50^,^51^ or neofunctionalization^52^ has been invoked to account for preservation of ancient duplicate genes including zebrafish circadian clock genes, *clock*, *bmal* and *period*^21^,^22^,^23^. Our type I functional divergence analyses detected that the site-specific selective constraints between each pair of *Cry* paralogs *Cry1*/*Cry3*, *Cry1*/*Cry2*, or *cry1a*/*cry1b* were altered (p < 0.01) (Tables 1, 2), which could be construed as evidence for either subfunctionalization or neofunctionalization of these *Cry* duplicates. Furthermore, zebrafish *cry1ba/cry1bb* generated by the TGD also showed the asymmetric evolutionary rate (Supplementary Table S3). Specifically, zebrafish *cry1ba* had significantly higher numbers of unique sites than zebrafish *cry1bb*, implying that the former has undergone relaxed functional constraint, while the latter might have retained the original rate of evolution. Asymmetric evolutionary rates between duplicates also were regarded as evidence for either neofunctionalization^49^ or subfunctionalization^52^. Importantly, zebrafish *cry1aa*, *cry1ab*, *cry1ba*, *cry1bb*, *cry2* and *cry3* exhibit distinct patterns of temporal and spatial expression (Fig. 5 and Supplementary Fig. S5). The four *cry1* genes have also diverged from each other, for example, while the expression of *cry1ab* is under the control of the Clock:Bmal heterodimer, *cry1aa* is light-dependent^53^; and the NLS motif of Cry1bb is different from those of the other three Cry1 proteins. Nevertheless, these expression data provided strong evidence for tremendous divergence and evolution of the *cry* duplicates following the duplication.

### Two types of zebrafish Cry: repressive Cry (RC) and non-repressive Cry (NRC)

Fruit flies have one CRY protein that cannot repress CLOCK:CYCLE-mediated transcription and that serves as a blue-light photoreceptor for photic entrainment^16^,^17^, while mice have two light-independent CRY proteins (mCRY1 and mCRY2) that function in the mammalian central clockwork as potent repressors of CLOCK:BMAL1-mediated transcription^5^,^13^. Genome interrogation and phylogenetic analysis recently revealed that at the base of the metazoan radiation, gene duplication as well as gene losses generated two types of insect Cryptochromes: a Drosophila-like CRY1 (dlCRY1) that is photosensitive but non-repressive and a mouse-like CRY2 (mlCRY2) that is repressive but photo-insensitive^54^,^55^. Among insects, flies have only dlCRY1, mosquitos (*Anopheles gambiae*) and monarch butterflies (*Danaus plexippus*) have both dlCRY1 and mlCRY2; while honey bees (*Apis mellifer*a) and red flour beetles (*Tribolium castaneum*) have only mlCRY2 (Supplementary Fig. S8)^54^,^55^. As such, three distinct clockwork models have been proposed to operate in insects: Type 1 (or fly model) in which only CRY1 acts as a photoreceptor; Type 2 (or butterfly model) in which both dlCRY1 and mlCRY2 exist with the former being a photoreceptor and the latter being a transcriptional repressor; and Type 3 (or beetle model) in which only mlCRY2 functions as a transcriptional repressor^54^. Importantly, it appears that repressive but light-insensitive mlCRY2 is evolutionarily derived from their light-sensitive but non-repressive dlCRY1^54^. Moreover, our phylogenetic analysis of insect *Cry* genes showed that insect *dlCRY1* genes form a sister clade with vertebrate *Cry3* genes, while insect *Cry2* genes appear more similar to vertebrate *Cry1* and *Cry 2* genes (Supplementary Fig. S8).

In addition to mice and humans, other vertebrates including African frogs (*Xenopus laevis*) and domestic chicken (*Gallus gallus*) have been shown to possess two repressive CRY proteins^56^,^57^. Our results and the other study^29^ showed that zebrafish as a primitive vertebrate possess six Cry proteins that can be divided into two types, Repressive Cry (RC) and Non-Repressive Cry (NRC) (Fig. 6, Supplementary Fig. S5). Zebrafish RCs, Cry1aa, Cry1ab, Cry1ba and Cry1bb, are able to repress Clock:Bmal-mediated transcription; however, in all nine zebrafish Clock:Bmal combinations examined, zebrafish NRC Cry2 ( an ortholog of mammalian CRY2) and Cry3 (an ortholog of CRY3 in amphibians, reptiles and birds but gone missing from mammals) were found to have no effects on Clock:Bmal-mediated transcription (Fig. 6, Supplementary Fig. S3)^29^. In the phylogenetic tree, both Cry2 and Cry3 appear to diverge earlier (Fig. 1). We think that as in insect evolution^54^, the repressive function of the four zebrafish Cry1 proteins also is a derived state. Because these four zebrafish *cry1* genes have evolved the repressive function for circadian regulation, zebrafish *cry2* (the ortholog of mammalian *Cry2*), which lacks the repressive function, has been relaxing to evolve novel functions that remain to be unidentified. Similarly, zebrafish *cry3* had not evolved the repressive function, either, but could be a photoreceptor that requires further functional investigation^29^,^58^.

### Mechanistic explanations of non-repression of Cry2 and Cry3

*Cry* is an ancient gene that derived from DNA photolyase but lost the DNA repair function during the evolution^59^. To evolve the repressive circadian function, a Cry protein evolved the abilities to enter the nucleus (an NLS motif) and to interact with Clock or Bmal protein (a protein-protein interaction domain). We used a bioinformatic program to uncover that zebrafish the four repressive Crys (RCs), Cry1aa, Cry1ab, Cry1ba, Cry1bb and the zebrafish NRC Cry2 all possess predicted NLS motifs (Supplementary Fig. S4). Our *in vitro* cell assays showed that zebrafish RC Cry1bb is able to enter the nucleus, while only a portion of the population of zebrafish NRC Cry2 molecules can enter the nucleus, and zebrafish NRC Cry3 is located only in the cytoplasm (Fig. 6). Co-IP assays showed that Cry2 cannot bind to the Bmal1a protein as effectively as Cry1bb (Figs. 8A–D). Thus, it appears that although zebrafish Cry2 has evolved an NLS motif that allows it to enter the nucleus partially, it hasn't evolved an effective protein-protein interaction domain that is required for the repressive function. Zebrafish Cry3 hasn't yet evolved an NLS motif (Fig. 6E) and it hasn't evolved a required protein-protein interaction domain, either, because our construct that was translocated into the nucleus driven by strong NSL sequences still did not repress Clock:Bmal-mediated transcription (Figs. 8E and F).

What are the functions of zebrafish Cry2 and Cry3? Recent studies suggested that Cry proteins are likely to function as light-dependent magnetic field sensors, used by migratory insects, birds or fish for directional responses^60^,^61^,^62^. For example, Cry3 in migratory garden warbler (*Sylvia borin*) is recently suggested to serve as its magnetoreceptor^63^. The fact that zebrafish *cry2* and *cry3* are both rhythmically expressed like other four zebrafish *cry1* genes suggests that their undiscovered functions still may be related to circadian rhythmicity.

## Methods

### Phylogenetic, splice site and conserved syntenic analyses

The teleost fish *cry* genes and *Cry* genes of humans (*Homo sapiens*), coelacanths (*Latimeria chalumnae*), zebra finches (*Taeniopygia guttata*), western clawed frogs (*Xenopus tropicalis*), anoles (*Anolis carolinensis*), chicken (*Gallus gallus*) and fruit flies (*Drosophila melanogaster*) were uncovered from Ensembl (http://www.ensembl.org/index.html). The Ensembl ID numbers of these genes are listed in Supplementary Table S1. Multiple sequence alignments of the CRY peptides were generated using Clustal X^64^. The tree was constructed by Maximum Likelihood (ML) with MEGA6^65^. The phylogeny was tested by the bootstrap method with 500 replications. The substitution model was the Dayhoff model. Numbers indicate bootstrap values.

Exon boundaries of the coding regions within *cry* genes were determined according to Ensembl. The numbers of nucleotides (nt) for each exon as well as the phase of each slicing site were also determined and are shown in Fig. 2. Using chicken *Cry1* (Gga1), *Cry2* (Gga5) and *Cry3* (Gga26) as anchor sites for conserved syntenic analysis of teleost fish *cry1, cry2* and *cry3*, the orthologous comparisons of the genes in the regions flanking the chicken *Cry* loci (goGor3) with the zebrafish genome (Zv9), the fugu genome (FUGU4.0), the tetraodon genome (TETRAODON8.0), the medaka genome (HdrR), the stickleback genome (BROADS1) were conducted with the BioMart mode in Ensembl, respectively. From the BioMart output tables, only genes that exhibit a 1 to 2 (human/chicken to zebrafish/fugu/tetraodon/medaka/stickleback) orthology that were supported by phylogenetic analysis (data not shown) were chosen. The Ensembl gene ID numbers and the genomic locations of zebrafish, medaka, stickleback, coelacanths, chicken, anoles and humans are shown in Supplementary Table S2.

### Analysis of type I functional divergence and test of relative evolutionary rates

Type I functional divergence after gene duplication leads to altered selective constraints between duplicate genes. The DIVERGE program^38^,^39^ was employed to examine whether one of the duplicates has evolved at an accelerated rate following the duplication. Tajima relative rate tests^43^ ere conducted with amino acid sequences for the four fish *cry* duplicate pairs using MEGA6^65^.

### Zebrafish maintenance and embryo production

The zebrafish wild-type AB strain is maintained at the Soochow University Zebrafish Research Facility as described previously^66^. Wild-type embryos were produced by pair matings, collected for RNA extraction, and fixed for *in situ* hybridization experiments at specified stages.

### RNA extraction and quantitative real-time PCR (qRT-PCR)

Total RNAs were extracted from zebrafish larvae at specific time points with TRIzol reagent (Invitrogen). First-strand cDNAs were synthesized from total RNAs by reverse transcription using the Invitrogen SuperScript First-Strand Synthesis System for RT-PCR according to manufacturer's instructions. Quantitative RT-PCR was performed with an ABI StepOnePlus system and SYBR Green qRT-PCR Master Mix (Invitrogen). Primer sequences used for *cry* genes and *actb1* as an internal control are listed in Supplementary Table S4. All reactions were performed in triplicate.

### Generation of RNA probes and whole-mount *in situ* hybridization

The DNA templates for generating RNA probes were amplified from zebrafish larval RNAs using primers listed in Supplementary Table S4 as described previously^67^. RT-PCR products were then subcloned into the pEASY-T3 vector (Transgene). RNA probes were labeled with Digoxigenin (DIG) using an RNA labeling kit (Roche). Whole-mount *in situ* hybridization was conducted as previously described^67^. In short, fixed larvae were incubated in 50% formamide hybridization buffer with a DIG-labeled RNA probe at 70°C for 18 to 20 h. Both NBT (nitro blue tetrazolium) and BCIP (5-bromol-4-chloro-3-indolyl phosphate) (Roche) were used for colorimetric detection. For each *in situ* hybridization experiment, 10 to 15 larvae were used.

### Image acquisition and analysis

*In situ* hybridization images were acquired with a Leica M165 FC stereomicroscope and a digital camera and processed with Image Pro Plus (Media Cybernetics, Bethesda, MD) and Adobe Photoshop (San Jose, CA).

### Plasmid construction

The *per2* promoter reporter [*per2*-luc (luciferase)] was constructed by inserting an approximately 450 bp region of the zebrafish *per2* promoter containing one E-box and three E'-box into the pGL3 basic vector^68^. Three *bmal* genes, three *clock* genes and six *cry* genes were cloned from zebrafish wild-type brain and eye cDNAs as templates. These clones were inserted into pMD19T vector (TAKARA), and then subcloned into pcDNA3.1. Each constructed plasmid was confirmed by DNA sequencing. All primers used for cloning and site-directed mutagenesis are listed in Supplementary Table S4.

### Cell culture and transfection

Co-transfection experiments were performed with HEK-293T cells (human embryonic kidney 293T cells). HEK293 cells were cultured in Dulbecco modified Eagle medium (DMEM) supplemented with 10% fetal bovine serum (Hyclone) under 5% CO_2_ at 37°C. Plasmid transfections were performed in 24-well plates (3 × 10^4^ cells/well). One day after seeding, cells reached 90%–95% confluency, the medium was changed and transfection mixture was added to each well. The cells were transfected using Lipofectamine2000 (Invitrogen) according to the manufacturer's protocol. Each transfection contained the *per2*-luc (10 ng), pRL-null *Renilla* plasmid (1 ng), zebrafish *clock* and *bmal* (each at 150 ng per transfection), and *cry* (each at 250 ng). The total amount of DNA per well was adjusted to 650 ng by adding pcDNA 3.1 vector as carrier. After 5–6 h, the transfection mixture was replaced with a fresh DMEM culture medium containing 10% bovine serum and after an additional 24 h, cells were lysed (passive Lysis Buffer, 150 uL; Promega) and luciferase activities were determined with a Luminoskan Ascent Microplate Luminometer (Thermo Scientific). The firefly luminescence signal was normalized based on the *Renilla* luminescence signal. Each of the transfections was performed in triplicate.

### Antibodies and immunoprecipitation

Anti-Cry1ab, anti-Cry2 and anti-Bmal1a polyclonal antibodies were raised in rabbits by using partial fragments of zebrafish Cry1ab (Transcript ID: ENSDARG00000011583, Protein ID: ENSDARP00000104471, Location of antigen: aa589–602), Cry2 (Transcript ID:ENSDART00000123497, Protein ID:ENSDARP00000108464, Location of antigen: aa585–598) and Bmal1a (Transcript ID:ENSDART00000023959, Protein ID:ENSDARP00000004223, Location of antigen: aa539–553) as antigens, respectively. Immunoprecipitation was performed as described previously^69^. Briefly, HEK293 cells were seeded in 6-cm dishes, and were transfected the following day with expression plasmids. Cells were washed twice with phosphate-buffered saline (PBS) 24 h after transfection, homogenized in binding buffer (150 mm NaCl, 5 mm EDTA, 0.5% Nonidet P-40, and 50 mm Tris-HCl, pH 7.5) containing protease inhibitor mixture Tablets, and then clarified by centrifugation for 10 min at 10,000 × g. Total protein (100 μg) from the supernatant was incubated with 15 μl of protein A/G-agarose beads (Santa Cruz) for 1 h at 4°C, and then centrifuged. The supernatant was incubated for 12 h at 4°C with the anti-Bmal1a or anti-Cry1ab, and 15 μl of protein A/G-agarose beads. The beads were then washed three times with binding buffer, boiled in SDS sample buffer, and centrifuged. The supernatant was separated by SDS-PAGE and analyzed by Western blotting. Intensities of bands were quantified with ImageJ (National Institutes of Health).

### Fluorescent microscopy

48 h after transfection, cells were stained with Hoechst 33258 (10 μg/ml) (Sigma-Aldrich) at 37°C for 10 min. EGFP and Hoechst fluorescence was visualized using an Olympus (IX70) fluorescent microscope and images were captured with a Spot digital camera (RTke, Diagnostic instruments, MI, USA).

### Statistical analysis

Results were analyzed by either Student's *t*-test or ANOVA, and data are presented as mean ± SD (standard deviation) with significance level at p < 0.05.

## Supplementary Material

Supplementary Information Supplementary information

Supplementary Information

Supplementary Information

## Figures and Tables

**Table 1:** Type I functional divergence for *Cry* genes

Groups	θ ± SE	*P*	0.9 > Qk > 0.67	Qk > 0.9
CRY1 and CRY2/Cry3-fish + tetrapod	0.495 ± 0.055	*P* < 0.01	34	11
CRY1/ CRY2	0.341 ± 0.055	*P* < 0.01	7	3
Cry1a/Cry1b	0.395 ± 0.106	*P* < 0.01	12	0
Cry1a/Cry1-tetrapod	0.510 ± 0.140	*P* < 0.01	19	0
Cry1a/Cry2-tetrapod	0.335 ± 0.085	*P* < 0.01	12	0
Cry1a/Cry2-fish	0.376 ± 0.098	*P* < 0.01	28	0
Cry1a/Cry3-fish + tetrapod	0.497 ± 0.076	*P* < 0.01	74	3

Functional divergences (θ) for pairwise comparisons of the Cry proteins of teleost fish are shown as value ± standard error. 327 sites were investigated based on posterior probability (Qk) within Cry proteins.

**Table 2:** Site of Type I functional divergence

Groups	0.9 > Qk > 0.67	Qk > 0.9
Cry1a/Cry1b -fish	92K,94N,114K,125I,137K,162A,165T,179T,219T,305R,311G,479L	
Cry1a/Cry1- tetrapod	125I, 179T, 183S, 203E, 248Y, 392M, 437R, 441H, 454V	
Cry1a/Cry2- tetrapod	118N, 126V, 128I, 137K, 162A, 168E, 180T, 181P, 189K, 211P, 311G, 461I	
Cry1a/Cry2- fish	93I,94N,114K,118N,128I,137K,161E,162A,172A,174V,181P,183S,189K,201D,203E,211P,222E,233N,236R,237P,239M,241A,265F,281S,305R,459K,472H,479L,	
Cry1a/Cry3 Fish + tetropod	53R,57Q,58C,63D,65S,67R,79Q,82D,83V,89K,92N,93I,100Y,111A,112A,115K,116L,120A,121G,128I,137K,139I,140E,146S,157I,159R,161E,173E,174V,180T,183S,189K,190F,193P,194S,196E,208A,209V,211P,216E,223R,225L,226E,227R,234F,235E,247S,264L,271D,274R,281S,283P,299A,314I,318I,319P,324P,338P,372I,373S,378M,386L,408Q,417S,427D,428Y,443Y,454V,456K,459K,461I,470M,482E,485K,	94, 305, 311

148 sites were investigated based on posterior probability (Qk) within CRY proteins. Sites with 0.9 > Qk > 0.67 or Qk > 0.9 are listed relative to zebrafish Cry1aa protein sequence. Sites located at N-terminal regions RD-2a, RD-1 or RD-2b are high-lighted in yellow, green or blue, respectively.
