# Supplementary material for: Molecular evolution and functional divergence of zebrafish (Danio rerio) cryptochrome genes
Source: Sci Rep. 2015 Jan 29;5:8113. doi: 10.1038/srep08113 (PMC4558521; doi:10.1038/srep08113)
Supplement: Supplementary Information [file srep08113-s1.doc]

**Supplementary information**

**Molecular evolution and functional divergence of zebrafish (*Danio rerio*) *cryptochrome* genes**

Chao Liu^1,2^*, Jia Hu^1,2^*, Chunxiang Qu^2^*, Lin Wang^3^, Guodong Huang^1,2^ , Pengfei Niu^1,2^ , Zhaomin Zhong^1,2^, Fashui Hong^2^, Guanghui Wang^4^, John H. Postlethwait^5^, and Han Wang^1,2,§^

^1^Center for Circadian Clocks, Soochow University, Suzhou 215123, Jiangsu, China

^2^School of Biology & Basic Medical Sciences, Medical College, Soochow University, Suzhou 215123, Jiangsu, China ^3^School of Computer Science and Technology, Soochow University, Suzhou 215006, Jiangsu, China ^4^College of Pharmaceutical Sciences, Soochow University, Suzhou 215123, Jiangsu, China ^5^Institute of Neuroscience, 1254 University of Oregon, Eugene, OR 97403, USA.

*These authors contributed equally to this work

^§^ Correspondence and requests for materials should be addressed to H.W. (han.wang88@gmail.com; wanghan@suda.edu) Tel: +86 51265882115; Fax: +86 51265882115

Current address for Fashui Hong: School of Life Science, Huaiyin Normal University, Huaian 223300, China.

**Supplementary Tables**

**Supplementary Table S1 The *cry* genes used for phylogenetic analyses** (See Excel file)

**Supplementary Table S2 The genes used for conserved syntenic analyses** (See Excel file)

**Supplementary Table S3 Results of Tajima relative rate tests of fish *cry* genes as duplicate pairs compared to their human orthologues**a

| Testing group | Mt^b^ | M1 ^c^ | M2 ^d^ | χ^2^ | *P* ^e^ |
| --- | --- | --- | --- | --- | --- |
| *cry1aa_Dr vs . cry1ab_Dr with Cry1_Xt* | 504 | 16 | 26 | 2.38 | 0.123 |
| *cry1ba_Dr vs . cry1bb_Dr with Cry1_Xt* | 477 | 66 | 21 | 23.28 | 0.000 |
| *cry1aa_Ga vs . cry1bb_Ga with Cry1_Xt* | 432 | 33 | 29 | 0.26 | 0.611 |
| *cry1aa_Ol vs . cry1bb_Ol with Cry1_Xt* | 452 | 19 | 43 | 9.29 | 0.002 |

^a^ The equality of evolutionary rate between a fish duplicate pair is tested using *Xt*, *Xenopus tropicali* orthologue as an outgroup using the Tajima relative rate test with amino acid sequences. *Dr*, *Danio rerio*; *Ol*, *Oryzias latipes*; *Ga*, *Gasterosteus aculeatus*.

^b^ Mt is the sum of the identical sites and the divergent sites in all three sequences tested.

^c^ M1 is the number of unique differences in the first fish paralogue.

^d^ M2 is the number of unique differences in the second fish paralogue.

^e^ If *P*<0.05, the test rejects the equal substitution rates between the two fish duplicates and infers that one of the two duplicates has a faster evolutionary rate.

**Supplementary Table S4 PCR primers**

| **Gene** | **Forward primer** | **Reverse primer** | **Accession no.** | **note** |
| --- | --- | --- | --- | --- |
| ***bmal1a*** | TGCTTGAATGAATCTGTGCGG | TGGCTCCCGTTTGCCTTG | NM131577.1 | PCR |
| ***bmal1b*** | AGGAAAGTAGGTCATAGCGAATGT | CACAGTAACAAAGAGTCATGATTCC | ENSDART00000098259 | PCR |
| ***bmal2*** | ATGTCGTCGAGGAACACAGC | TCAGAGAGACCAGTGCATCTCGT | NM131578.1 | PCR |
| ***cry1aa*** | GAGAGTGTTGTAGAATAACCCTTGC | CCCGAGGACAGGCTCAGTA | ENSDARG00000045768 | PCR |
| ***cry1ab*** | ATGGTTGTCAATACGGTCCACT | ACGACAAAGCGTGTTCCTCATC | ENSDARG00000011583 | PCR |
| ***cry1ba*** | ATGGCCCCAAATTCCATC | TTAACTCCTCATACTTGACTGATTC | ENSDARG00000091131 | PCR |
| ***cry1bb*** | GGACTTTATTGGACTGAGGTG | AAATTGTTTAGACGCAATCTG | ENSDARG00000069074 | PCR |
| ***cry2*** | TCCTTGGAACTTTTACGATG | TCCGACTCGCTTGGTTATG | ENSDARG00000024049 | PCR |
| ***cry3*** | AAGTGTCCTAATGGATGTTGC | TCTCCGCTGAGCTCTGATA | ENSDARG00000011890 | PCR |
| ***clock1a*** | ATGACCTCCAGCATAGACCG | TTACTGAGGCGGAGGGTTG | ENSDARG00000011703 | PCR |
| ***clock1b*** | GACTGATCCATGAGACCGAGTTAT | TTACTGAGACTGGGCACTAGCG | 3ENSDARG00000003631 | PCR |
| ***clock2*** | ATGGGAGAAGGAGTGAGCAT | CTACCGAGGGAGCATGTTTCT | NM178299 | PCR |
| ***cry1bb***  ***△1*** | GCTCTAGAACCCGGGGATCCTCTAGA | CCCAAGCTTAGAGTCCGCGGTATCTTGA |  | PCR |
| ***cry1bb***  ***△2*** | GCTCTAGAACCCGGGGATCCTCTAGA | CCCAAGCTTAAGACGATGCGTCGTTTCGT |  | PCR |
| ***cry1bb***  ***△3*** | GCTCTAGAGTGCTGCGCAGGTTCTTC | CCCAAGCTTATGGTCCCGGTTGCTCTCT |  | PCR |
| ***cry1bb***  ***△4*** | GCTCTAGACTGTGCTGCGCAGGTTCT | CCCAAGCTTAGTGTCGGTGTCGATGTTTG |  | PCR |

**Supplementary Figures:**

**
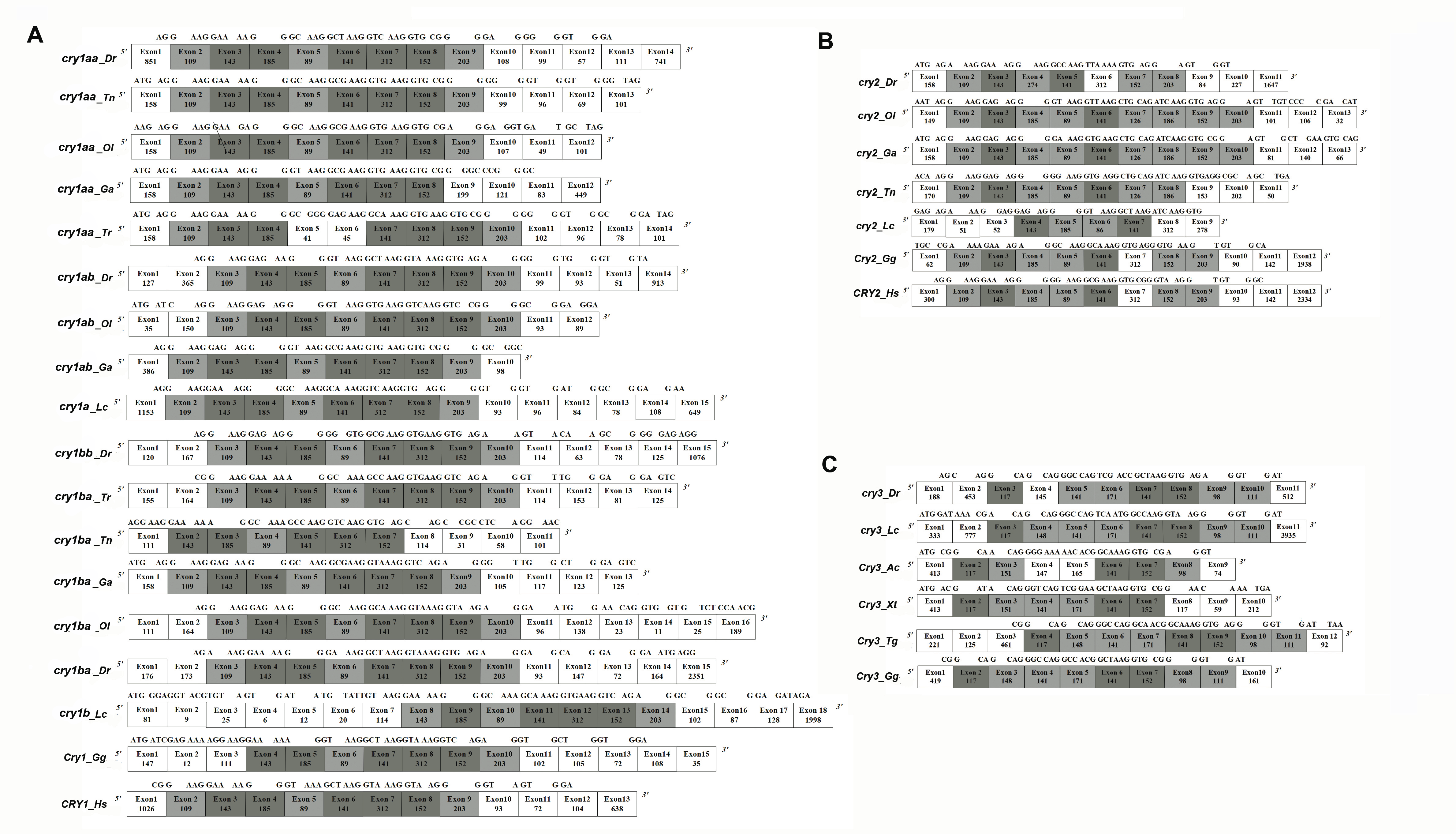
**

**Supplementary Figure S1. Exon/intron structures** **of *cry* genes.** (A) Exon/intron structure of teleost and coelacanths *cry1a* genes, chicken *Cry1* gene and human *CRY1* gene. (B) Exon/intron structures of teleost and coelacanth *cry2* genes, chicken *Cry2* gene and human *CRY2* gene. (C) Exon/intron structures of teleost, coelacanth, frog, anole, zebra finch and chicken *Cry3* genes. The sequences at the splice sites crossing or flanking exons are shown on top of each exon boundary. *Dr, Danio rerio; Tr, Takifugu rubripes; Tn, Tetraodon nigroviridis; Ol, Oryzias latipes; Ga, Gasterosteus aculeatus;* *Lc, Latimeria chalumnae*; *Hs, Homo sapiens; Gg, Gallus gallus; Tg, Taeniopygia guttata; Ac, Amoles carolinensis and Xt, Xenopus tropicalis*. Exon sizes are not drawn to scale. Dark grey columns indicate highly similar exons of the same size among different species and light grey columns similar exons among different species.

**
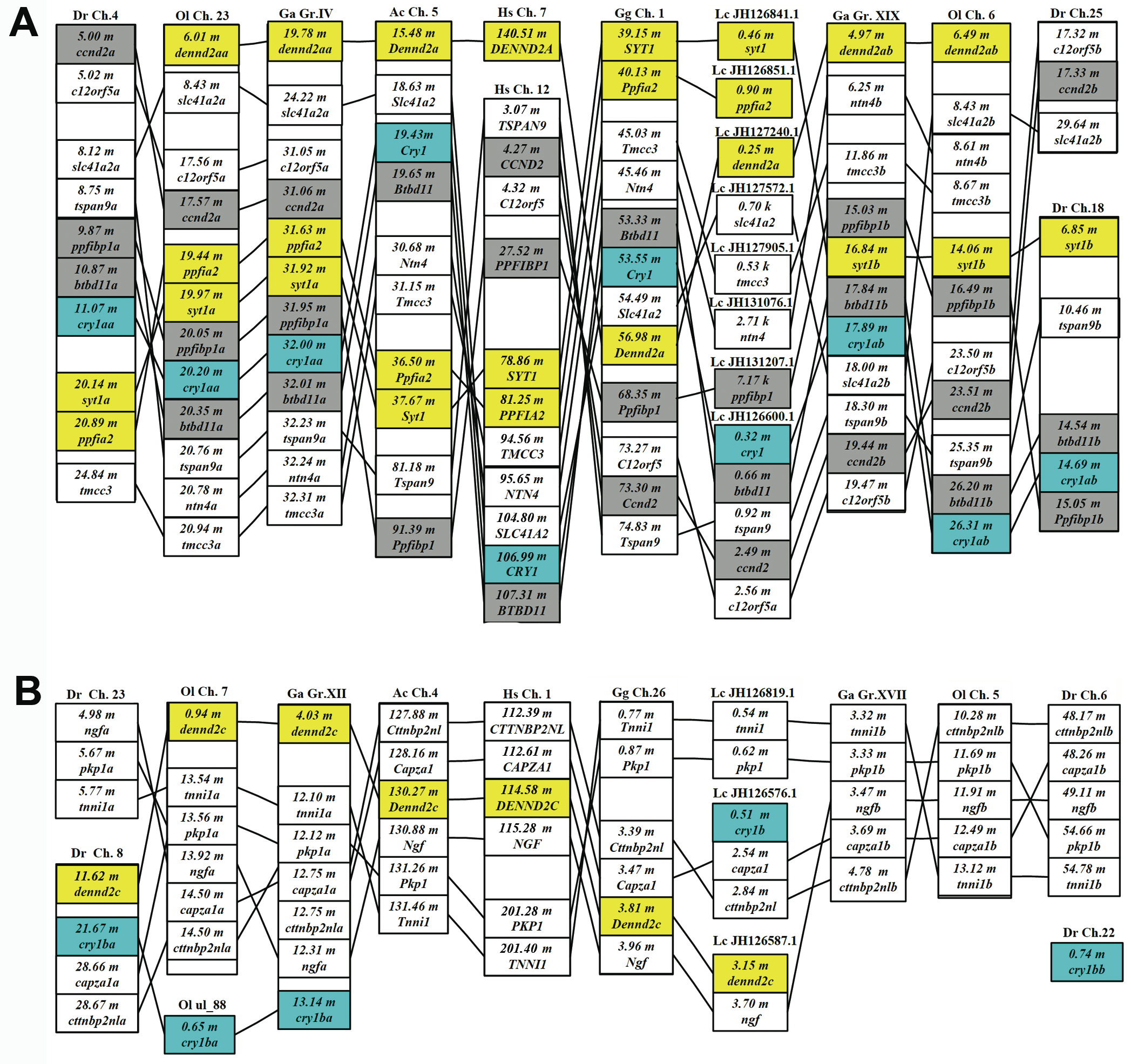
**

**Supplementary Figure S2. Comparison of gene orders surrounding *Cry1* in chromosomes of zebrafish, medaka, stickleback, coelacanths, chicken, anoles and human.** Zebrafish, medaka and stickleback each have two co-orthologs for a majority of these genes flanking *CRY1* on human chromosome 12 by using human *CRY1* as an anchor site (A). Using stickleback *cry1ba* as an anchor site, the orthologous genes of the genes linked to *cry1b* on fish were founded in human chromosomes (B). The gray column indicated paralogous pairs comprised human *CRY1* paralogon with the human *CRY2* paralogon. The yellow column indicated flanking paralogous pairs of *Cry1/Cry2/Cry3. Dr, Danio rerio; Ol, Oryzias latipes; Ga, Gasterosteus aculeatus; Hs, Homo sapiens; Tg, Taeniopygia guttata; Ac, Anolis carolinensis. Lc, Latimeria chalumnae.* The Ensembl ID numbers of these genes are listed in Supplementary Table S2. Abbreviations: m or k, million or kilo base pairs from one end of the chromosome, linkage group, or scaffold where the gene is located. The positions of genes on chromosomes are not drawn to scale.

**
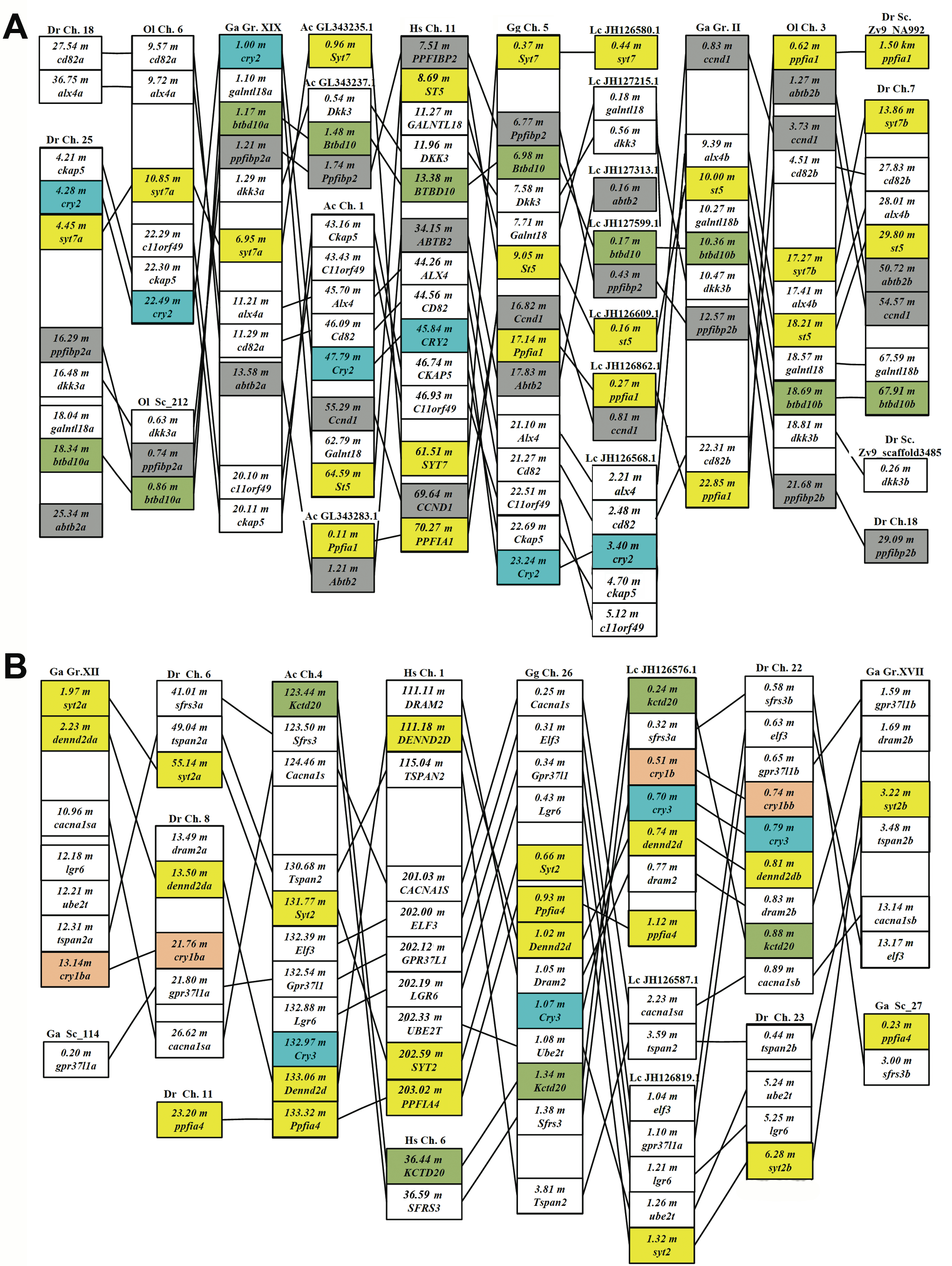
**

**Supplementary Figure S3. Comparison of gene orders surrounding *Cry2* or *Cry3* genes.** (A) Comparison of gene orders surrounding *CRY2* in chromosomes of zebrafish, medaka, stickleback, coelacanth, chicken, anole and human. The human *CRY2* region corresponds to two co-orthologous fish paralogons; one paralogon contains *cry2a*, the other paralogon lacks *cry2b* and some co-orthologs for genes flanking *CRY2* on Human chromosome 11. (B) Comparison of gene orders surrounding *cry3* and *cry1b* in chromosomes of zebrafish, medaka, stickleback, coelacanth, chicken, anole and human. Gray boxes indicate paralogous pairs comparing the human *CRY1* paralogon with the human *CRY2* paralogon. Yellow boxes indicate paralogous pairs of genes flanking. *Dr, Danio rerio; Ol, Oryzias latipes; Ga, Gasterosteus aculeatus; Hs, Homo sapiens; Tg, Taeniopygia guttata; Ac, Anolis carolinensis; Lc, Latimeria chalumnae*. Ensembl ID numbers of these genes are listed in Supplementary Table 2. Abbreviations: m or k, million or kilo base pairs from one end of the chromosome, linkage group, or scaffold where the gene is located. Positions of genes on chromosomes are not drawn to scale.

**
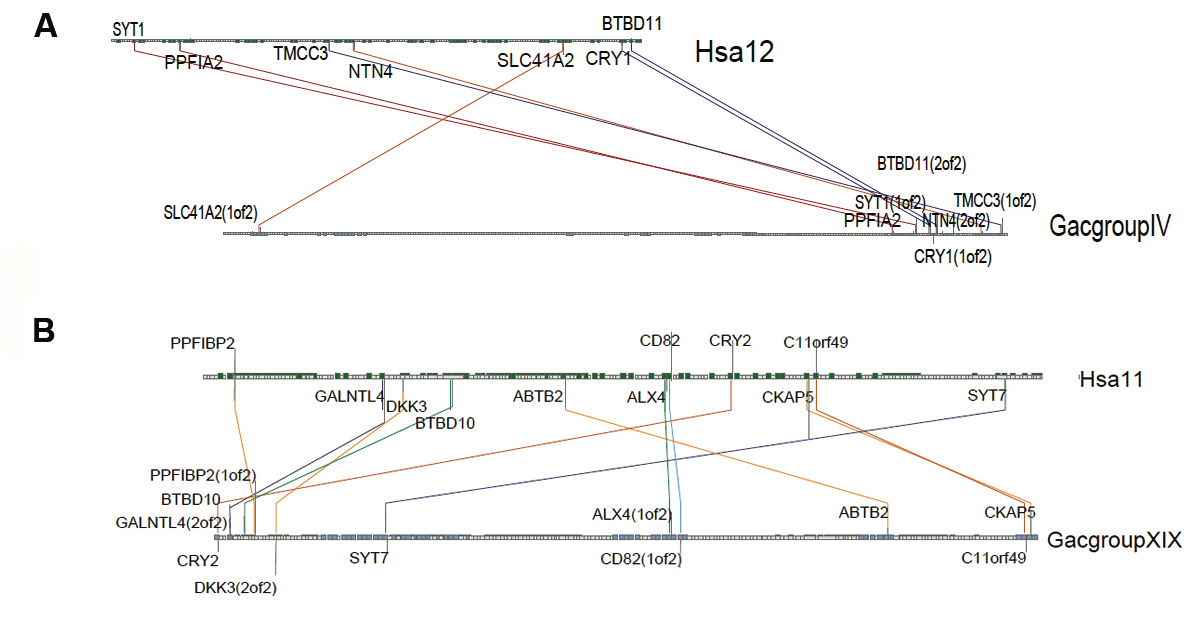
**

**Supplementary Figure S4.** **Comparison of the orders of paralogous genes surrounding *CRY* on human chromosome to the stickleback genome using the synteny database (http://syntenydb.uoregon.edu/synteny_db/) ^33^**. (*A*) Comparison of the orders of paralogous genes surrounding *CRY1* on human chromosome (Hsa12) to the stickleback genome (GacgroupⅣ).(*B*) Comparison of the orders of paralogous genes surrounding *CRY2* on human chromosome 11 to the stickleback genome (Gacgroup XIX).


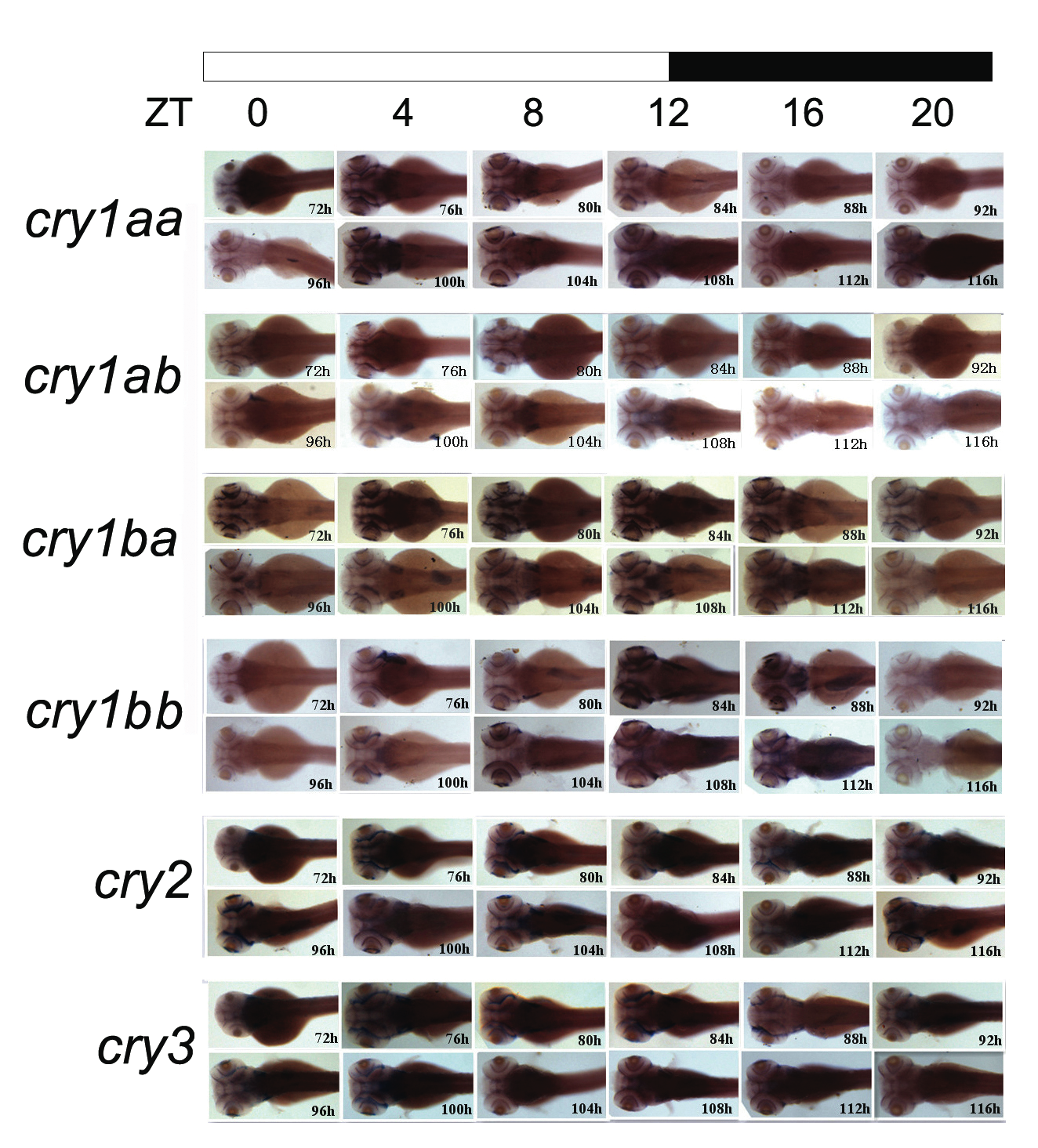


**Supplementary Figure S5. Dorsal views of expression patterns of zebrafish *cry1aa, cry1ab, cry1ba, cry1bb, cry2* and *cry3* shown by *in situ* hybridization**. Zebrafish *cry1aa, cry1ab, cry1ba, cry1bb, cry2* and *cry3* display diverse and rhythmic expression patterns in larvae. Whole-mount *in situ* hybridization was performed to examine expression patterns of six *cry* genes from 72 hpf to 116 hpf (Day 4 and Day 5, at 4 h intervals each day).

**
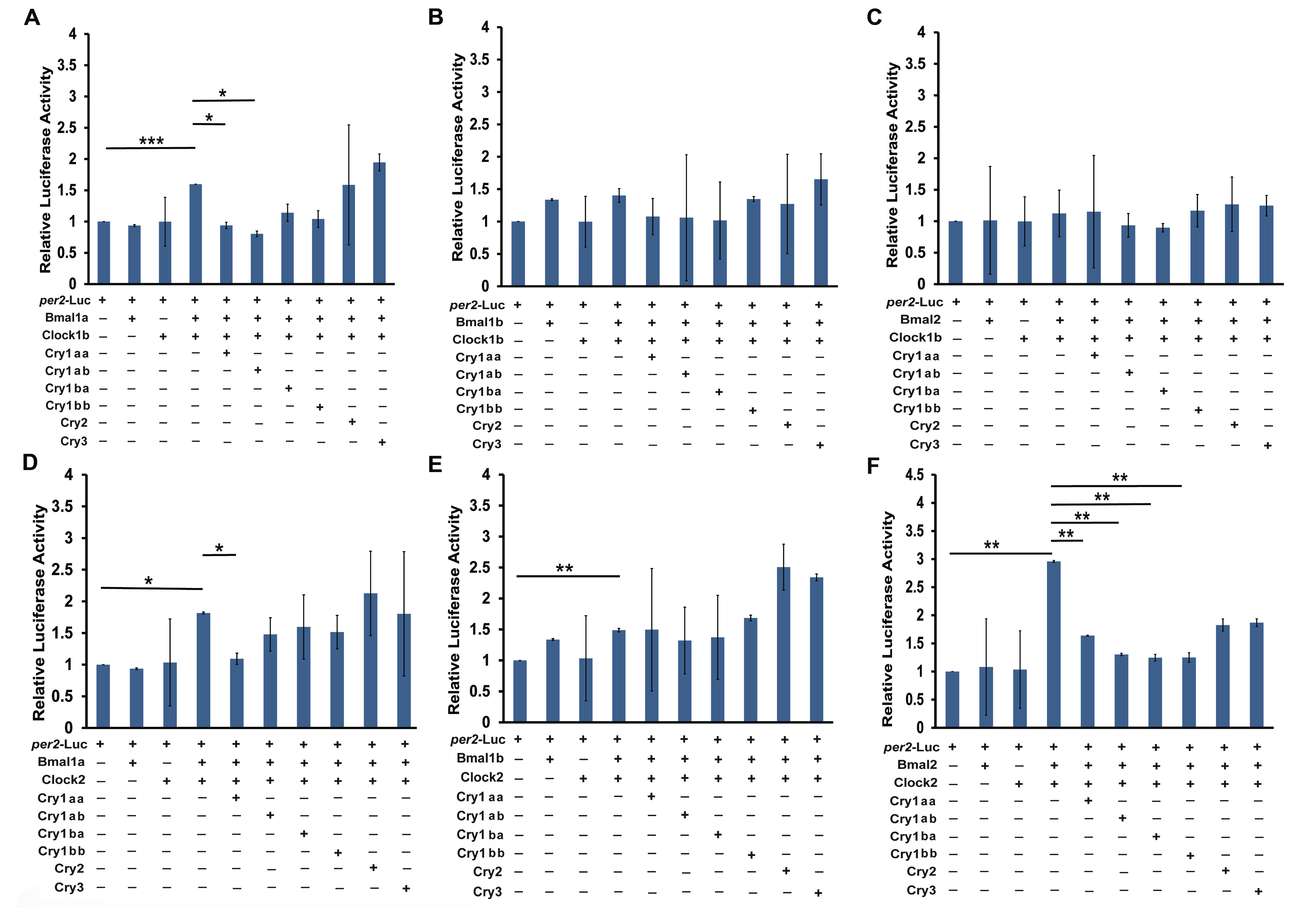
**

**Supplementary Figure S6. Repression of zebrafish Cry1 proteins on different Clock-Bmal combinations shown by luciferase reporter assays**. (*A*) Bmal1a:Clock1b combination. (*B*) Bmal1a:Clock2 combination; (*C*) Bmal1b:Clock1b combination, (*D*) Bmal1b:Clock2 combination, (*E*) Bmal2-Clock1b combination, (*F*) Bmal2:Clock2 combination. Each value is the mean±SEM of three independent experiments. Results were analyzed by ANOVA.

**
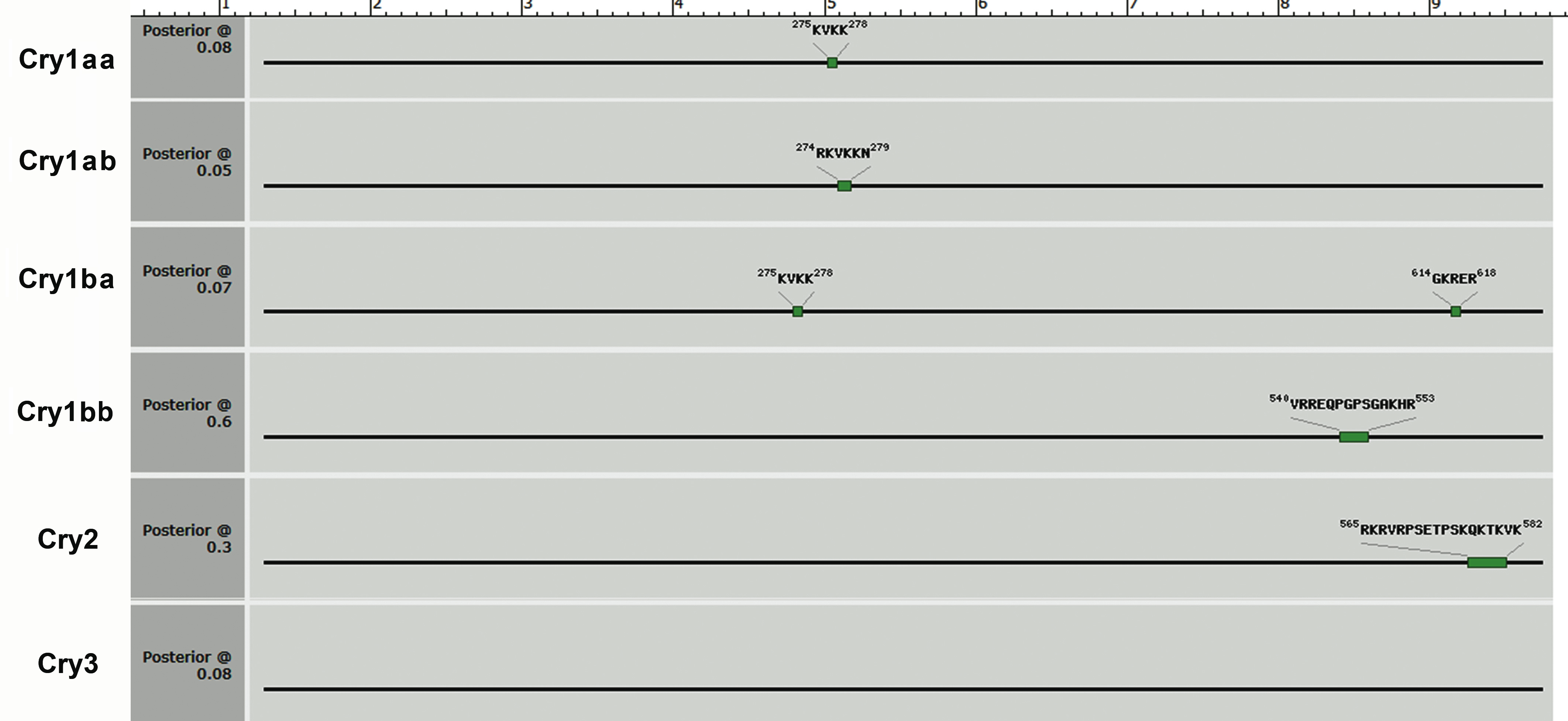
**

**Supplementary Figure S7.** **The predicted NLS sequences of zebrafish Cry proteins**. The NLS sequences of six zebrafish Cry proteins were predicted using www.moseslab.csb.utoronto.ca/NLStradamus ^46^.


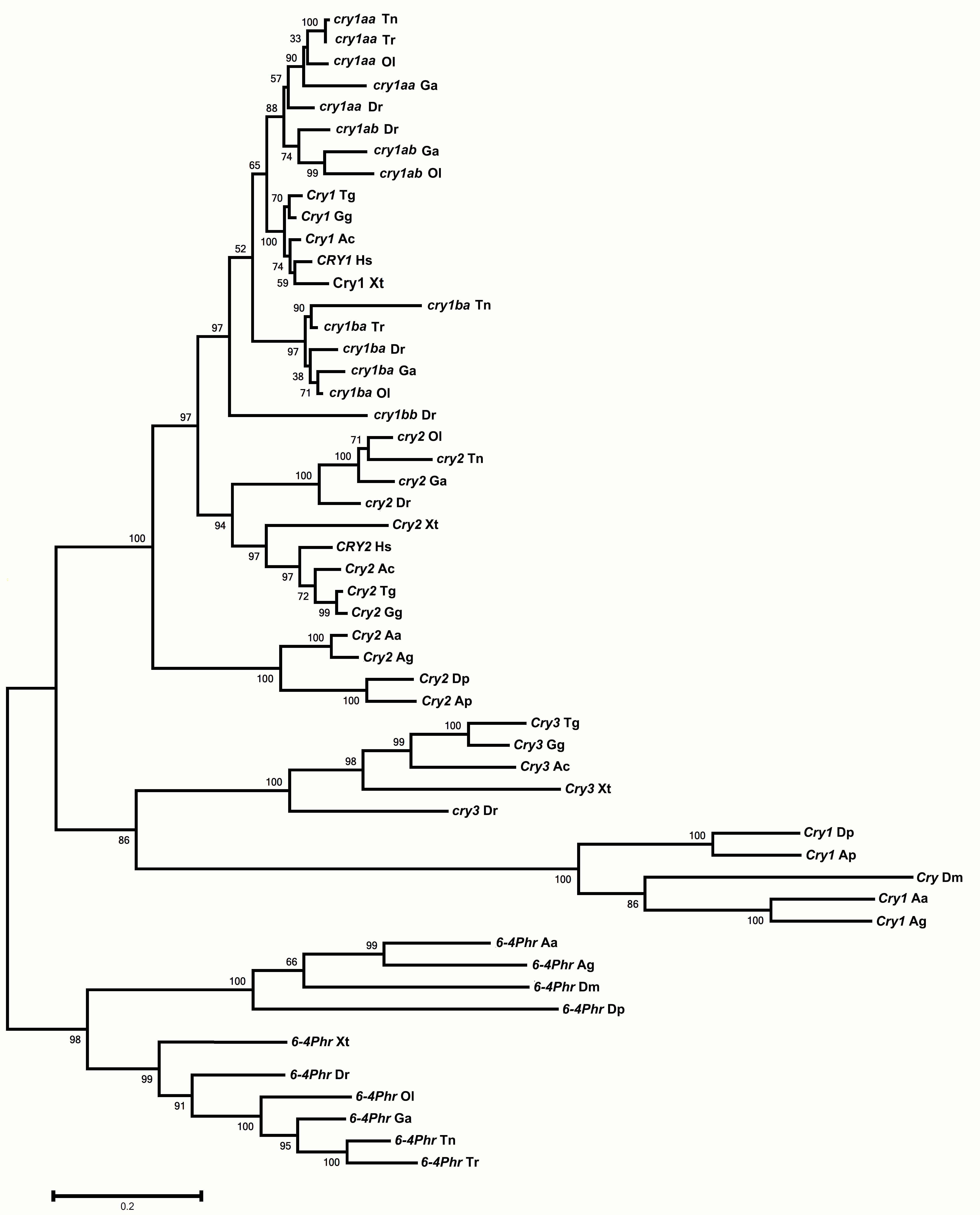


**Supplementary Figure S8. Phylogenetic analysis of insect and vertebrate *Cry* genes.** The tree was constructed by the Maximum Likelihood (ML) method using MEGA6 ^65^ with 500 bootstrap replications and the Dayhoff substitution model. Numbers on branches indicate bootstrap support values. *Dr, Danio rerio; Tr, Takifugu rubripes; Tn, Tetraodon nigroviridis; Ol, Oryzias latipes; Ga, Gasterosteus aculeatus; Hs, Homo sapiens; Gg, Gallus gallus; Tg, Taeniopygia guttata; Aa, Aedes aegypti; Ac, Anolis carolinensis; Ag, Anopheles gambiae; Xt, Xenopus tropicalis*, *Ap*, *Antheraea pernyi*; *Dp, Danaus plexippus;* and *Dm, Drosophila melanogaster.* The Ensembl or Genebank ID numbers of these genes are listed in Supplementary Table S1.
